# Maternal Benzophenone Exposure Impairs Hippocampus Development and Cognitive Function in Mouse Offspring

**DOI:** 10.1002/advs.202102686

**Published:** 2021-10-28

**Authors:** Fengzhen Cui, Qingfei Pan, Siyi Wang, Faming Zhao, Runxin Wang, Tingting Zhang, Yaying Song, Jun He, Haolin Zhang, Qiang Weng, Yang Jin, Wei Xia, Yuanyuan Li, Guo‐Yuan Yang, Winnok H. De Vos, Jean‐Pierre Timmermans, Shunqing Xu, Yaohui Tang, Xia Sheng

**Affiliations:** ^1^ Key Laboratory of Environment and Health Ministry of Education & Ministry of Environmental Protection School of Public Health Tongji Medical College Huazhong University of Science and Technology Wuhan 430030 China; ^2^ Department of Computational Biology St. Jude Children's Research Hospital Memphis TN 38105 USA; ^3^ Department of Neurology Wuhan Fourth Hospital/Pu'ai Hospital Tongji Medical College Huazhong University of Science and Technology Wuhan 430033 China; ^4^ Neuroscience and Neuroengineering Research Center Med‐X Research Institute and School of Biomedical Engineering Shanghai Jiao Tong University Shanghai 200030 China; ^5^ Department of Histology and Embryology School of Basic Medical Sciences Tongji Medical College Huazhong University of Science and Technology Wuhan Hubei 430030 China; ^6^ Laboratory of Animal Physiology College of Biological Sciences and Technology Beijing Forestry University Beijing 100083 China; ^7^ Department of Biosciences University of Oslo Oslo 0316 Norway; ^8^ Laboratory of Cell Biology and Histology University of Antwerp Wilrijk Antwerp 2610 Belgium; ^9^ µNEURO Research Excellence Consortium University of Antwerp Wilrijk Antwerp 2610 Belgium

**Keywords:** 4‐hydroxybenzophenone, endoplasmic reticulum stress, neurodevelopmental toxicity, p65, protein kinase R‐like ER kinase‐eIF2*α*

## Abstract

Benzophenones are widely supplemented in personal care products, but little is known about its neurodevelopmental toxicity. The previous epidemiological study discovered a negative correlation between maternal exposure to a benzophenone metabolite 4‐hydroxybenzophenone (4HBP) and child's neurodevelopment, yet the causal relationship and detailed mechanism remain to be defined. Here, it is reported that prenatal, but not postnatal, exposure to environmentally relevant level of 4HBP impairs hippocampus development and causes cognitive dysfunction in offspring mice. Transcriptomic analyses reveal that 4HBP induces the endoplasmic reticulum stress‐induced apoptotic signaling and inflammatory response in hippocampal neural stem cells. Mechanistically, 4HBP exposure activates protein kinase R‐like ER kinase (PERK) signaling, which induces CHOP, inhibits I*κ*B translation, and transactivates p65, thereby promoting inflammation and apoptosis on multiple levels. Importantly, genetic or pharmacological inhibition of PERK pathway significantly attenuates 4HBP‐induced NF*κ*B signaling and neurodevelopmental abnormalities in mice and in a human brain organoid model. The study uncovers the neurodevelopmental toxicity of BP and cautions its exposure during pregnancy.

## Graphical abstract

This study shows that maternal exposure to benzophenone and its major metabolite 4‐hydroxybenzophenone during pregnancy disrupt endoplasmic reticulum proteostasis and trigger inflammation in the offspring hippocampal neural stem cells, leading to cognitive dysfunction and neurodevelopmental toxicity. This phenomenon can be greatly ameliorated by inhibiting the protein kinase R‐like ER kinase signaling of the unfolded protein response.

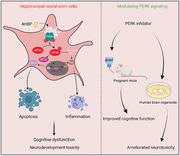

## 1 Introduction

Neural development is a complex process and exquisitelysensitive to environmental insults.^[^ ^1^ ^]^ Transcriptional programs in the fetal brain regulate complex and overlapping developmental processes that ultimately shape the functional architecture of the adult brain. Neural circuitry underlying higher brain functions, such as learning, is primarily established between the second trimester and early childhood,^[^ ^2^ ^]^ wherein the proliferation and differentiation of neural stem cells (NSCs) play a fundamental role. Exposure to environmental insults during this period of time may cause inflammatory response in NSCs, resulting in profound and long‐lasting adverse effects on neurodevelopment.^[^ ^3^ ^]^

The unfolded protein response (UPR) is an adaptive mechanism upon perturbations in the endoplasmic reticulum (ER), such as disrupted proteostasis.^[^ ^4^ ^]^ One of the canonical UPR sensors is the protein kinase R‐like ER kinase (PERK), which is able to phosphorylate Ser51 in the *α*‐subunit of eukaryotic translation initiation factor 2 (eIF2*α*). This transiently halts global translation, at the same time allows for translation of a small subset of mRNAs, such as ATF4. ATF4 elicits adaptive response by regulating the expression of genes involved in protein folding, autophagy, and redox homeostasis, while it also transactivates CHOP under chronic ER stress and triggers apoptosis.^[^ ^5^ ^]^ Crosstalk between ER stress and inflammation has been extensively studied in neuropathology,^[^ ^6^ ^]^ but their implication in neurodevelopmental disorders remains poorly understood.

Benzophenones (BPs) are widely used in personal care products owing to its protective effect on skin and hair from UV irradiation,^[^ ^7^ ^]^ and are generally considered as a safe supplement. The parental structure of BP comprises two aromatic rings and a carbonyl group, based on which a family of compounds has been derived, including 2,4‐dihydroxy‐BP (BP‐1) and 2‐hydroxy‐4‐methoxy BP (BP‐3).^[^ ^8^ ^]^ BP can be quickly metabolized into 4‐hydroxybenzophenone (4HBP), whose level peaks 4 h after oral administration in rats.^[^ ^9^ ^]^ 4HBP has been widely detected in human samples, including pregnant women's urine, serum, amniotic fluid, placenta, and breast milk.^[^ ^10^ ^]^ Several epidemiological studies have correlated 4HBP exposure with multiple disorders, including fecundity, urogenital malformation, and increased risk of diabetes.^[^ ^11^ ^]^ Of note, 4HBP has been shown to cross the placenta barrier,^[^ ^10b^ ^]^ yet its effect on neurodevelopment has not been characterized. Our previous study examined the maternal exposure level of three BP derivatives (4HBP, BP‐1, and BP‐3), where that of 4HBP showed a significantly negative correlation with impairment in child neurocognitive development.^[^ ^12^ ^]^ However, the causal relationship and underlying molecular mechanism remain to be elucidated.

In this study, we showed that maternal 4HBP exposure inhibited NSCs proliferation, which resulted in neuronal loss, impaired hippocampal development, and cognitive dysfunction in offspring mice. Interestingly, this phenotypic abnormality was not observed in adult mice exposed to the same dosage of 4HBP. Transcriptomic analysis of the 4HBP‐exposed NSCs identified evoked inflammatory response and ER stress‐mediated apoptotic signaling, to which activated PERK‐eIF2*α* branch of the UPR was found to be a central contributor. Strikingly, genetic or pharmacological inhibition of this signaling protected against the 4HBP‐associated neurodevelopmental abnormalities in mice and in a human neural organoid model. Together, our findings provide new insight into the mechanistic implications of neurodevelopmental toxicity induced by BPs exposure, and caution the use of BPs‐supplemented personal care products during pregnancy.

## 2 Results

### 2.1 Maternal 4HBP Exposure Impairs Cognitive Function in Offspring Mice

As described above, 4HBP is a major BP metabolite and was the only derivative we found with a robust correlation with child neurodevelopment,^[^ ^12^ ^]^ we thereby chose to use 4HBP instead of other BPs in our animal experiments to pinpoint its toxicity. Prior to investigating its effect on offspring cognitive function, we first determined the exposure dosage of 4HBP by treating pregnant mice to serial concentrations of 4HBP via drinking water throughout the pregnancy. Then, the level of 4HBP in serum, placenta, fetal brain was measured by Mass spectrometry, and compared to the concentrations reported in human studies by us and others.^[^ ^10^, ^11^, ^13^ ^]^ We found that the dose of 1 mg kg^–1^ day^–1^ 4HBP exposure fell close to environmentally relevant parameters (Figure S1a, Supporting Information), thus hereafter chose the dosage of 0.1 and 1 mg kg^–1^ day^–1^ 4HBP for the in vivo experiments in this study.

**Figure 1:**
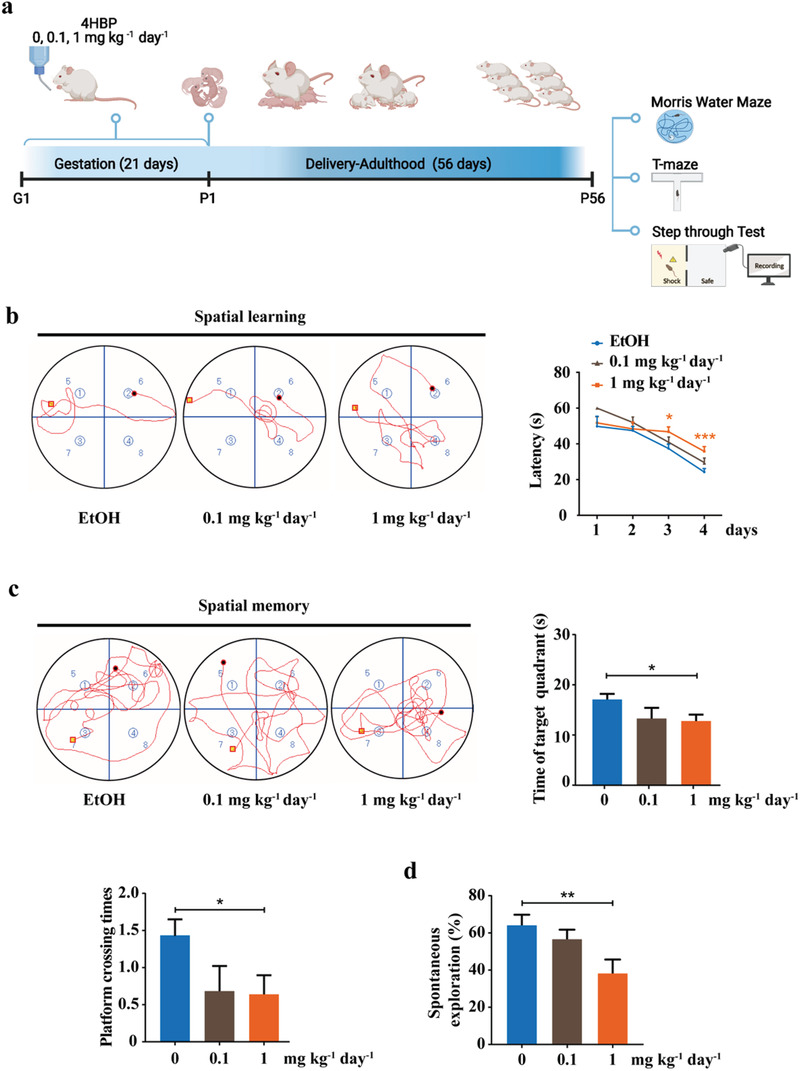
Maternal 4HBP exposure induces learning and memory defects in offspring mice. a) Schematic overview of the experimental design. Pregnant mice were exposed to either vehicle (EtOH) or 4HBP (0.1 or 1 mg kg^–1^ day^–1^) via drinking water throughout the entire pregnancy. The offspring were housed under normal condition from postnatal day 1 (P1) until day 56 (P56) for behavioral tests. b) On the left, representative swimming trace of mice from the start position to the platform in the spatial learning test on day 4. On the right, line graphs showed the change in escape latency from days 1 to 4 in the spatial learning tests. The hidden platform was fixed in the quadrant NE. c) Representative swimming trace in the spatial memory test after the hidden platform was removed on day 5. Bar graphs showed the time spent in the target quadrant and times of mice crossing the platform in spatial memory test among different groups. d) Quantification of spontaneous exploration behavior in the T‐maze. Data from *n* = 10 per group. Data are shown as mean ± SEM. One‐way ANOVA with Dunnett's multiple comparisons test, * *p* < 0.05, ** *p* < 0.01, *** *p* < 0.001. Parts of Figure 1a were made with BioRender.com.

Next, dams were exposed to 0.1 and 1 mg kg^–1^ day^–1^ 4HBP throughout pregnancy, and the offspring were housed under normal condition until postnatal day 56 (P56) when hippocampal development has matured, and synaptic connections and cognition‐related neural circuits have been stably established.^[^ ^14^ ^]^ Then, the mice were subjected to multiple behavior tests, including Morris water maze, step‐through test, and T‐maze test (**Figure** **1**a). In the water maze test, the escape latency gradually became shorter throughout the 4 days of spatial learning in all groups (Figure 1b). Spatial memory test on day 5 showed that 4HBP treatment reduced offspring swimming time in the target quadrant and the times of platform crossing, with statistically significant difference for 1 mg kg^–1^ day^–1^ exposure (Figure 1c). Meanwhile, the spontaneous exploration in the T‐maze test was significantly reduced in the 1 mg kg^–1^ day^–1^ 4HBP‐treatment group compared with the control groups (Figure 1d). Similarly, in the step‐through test we found that 4HBP exposure increased the time spent in and the number of repeated entries to the dark cage by the offspring (Figure S1b, Supporting Information). In contrast, 4HBP exposure of the same dosage and duration did not significantly alter the behavioral performance of adult mice (Figure S2a–c, Supporting Information), indicating a selective early‐life sensitivity to 4HBP exposure. These results indicate that maternal exposure to 4HBP impairs cognitive development in offspring mice.

### 2.2 Maternal 4HBP Exposure Disrupts Hippocampus Development in Offspring Mice

**Figure 2:**
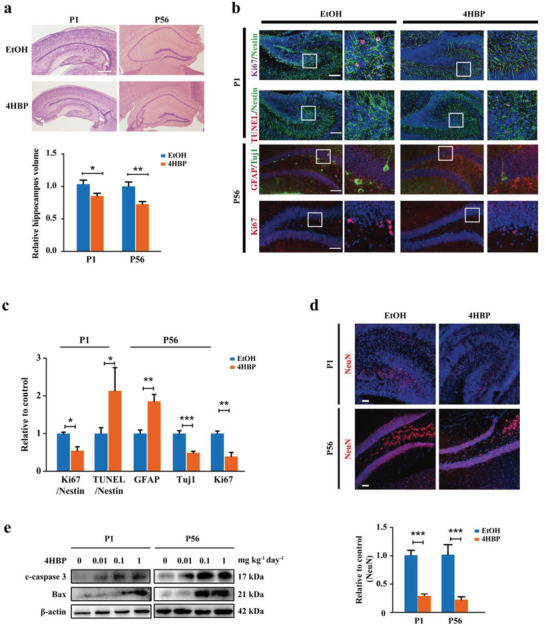
Maternal 4HBP exposure disrupts hippocampus development in offspring mice. Pregnant mice were exposed to either vehicle or 4HBP (1 mg kg^–1^ day^–1^) via drinking water throughout the entire pregnancy. The offspring were housed under normal condition until the indicated postnatal day. a) HE staining of the hippocampus and quantification of hippocampal volume in P1 and P56. b) Upper panel (P1): representative confocal images of NSCs co‐labeled with Nestin (green) and Ki67 (red) or TUNEL (red) in the dentate gyrus (DG) region of the hippocampus on P1. Lower panel (P56): representative confocal images of GFAP (green), Tuj1 (red), and Ki67 (red) staining in the DG of P56 offspring. Magnified images of the white box are shown to the right of each original image. Scale bar, 100 µm. c) Quantification of immunofluorescent staining in b. Data from *n* = 3 mice per group. d, top) Representative confocal images of NeuN in the DG region of the P1 and P56 hippocampus. Scale bar, 50 µm. Bottom, quantification of immunofluorescent staining in c. Data from *n* = 3 mice per group. e) Western analyses of cleaved‐caspase 3 and Bax in the hippocampal tissues of P1 and P56 offspring. Results of Western blot were from a representative experiment in triplicates. Data are shown as mean ± SEM. a,c,d) Unpaired and two‐tailed *t* test, * *p* < 0.05, ** *p* < 0.01, *** *p* < 0.001.

Next, we examined the effect of maternal 4HBP exposure on offspring hippocampus development. Hematoxylin & Eosin (HE) staining observed a significant decrease in the volume of 4HBP‐exposed hippocampus in P1, and the morphological difference remained in P56 (**Figure** **2**a). Immunofluorescent staining of P1 offspring hippocampal tissues showed that 1 mg kg^–1^ day^–1^ 4HBP exposure significantly reduced the number of Ki67^+^/Nestin^+^ NSCs and increased signal of TUNEL (Figure 2b,c). There was also a marked difference in the expression of GFAP and Tuj1 in the hippocampus of P56 offspring (Figure 2b,c). Interestingly, NeuN^+^ cells were significantly less, in particular in the P56 hippocampus, suggesting marked neuronal loss in the adult hippocampus (Figure 2d). In addition, elevated expression of cleaved‐caspase 3 and Bax was also detected in the hippocampal tissue lysates of 4HBP‐exposed offspring (Figure 2e). On the contrary, 4HBP exposure of the same dosage and duration did not significantly change the number of neurons or differentiation pattern in the hippocampus of adult mice (Figure S2d, Supporting Information), mirroring the results of the behavior test. Together, these observations authenticated the detrimental effect of early‐life 4HBP exposure on hippocampus development.

Next, we isolated NSCs from the hippocampus of E14.5 mice exposed to either control or 4HBP, and cultured to allow for neurosphere formation. The growth of 4HBP‐exposed neurospheres was significantly slower relative to controls (Figure S2e, Supporting Information). Consistently, 4HBP dose‐dependently inhibited viability and neurosphere formation of primary NSCs cultured in vitro (Figure S3a,b, Supporting Information). Of note, neither the parental BP nor BP‐3 caused significant toxicity at the same concentration (1 µM) (Figure S3a,b, Supporting Information), indicating that NSCs are significantly more vulnerable to 4HBP compared to these two common BPs, which is also in keeping with our previous epidemiological observation.^[^ ^12^ ^]^ Immunofluorescence observed decreased Ki67 and increased TUNEL signals in NSCs upon 1 µM 4HBP treatment (Figure S3c,d, Supporting Information), which was accompanied by higher levels of cleaved‐caspase 3 and Bax (Figure S3e, Supporting Information). These data implicate that 4HBP attenuates proliferation and induces apoptosis of hippocampal NSCs.

In the meantime, double staining of GFAP and Tuj1 in NSCs cultured under differentiation media revealed markedly increased GFAP and decreased Tuj1 signals in response to 1 µM 4HBP in vitro (Figure S4a, Supporting Information). Total dendritic length and terminal branch of neurons significantly reduced as compared to control group. Moreover, Sholl analysis revealed significant reduction in morphological complexity of Tuj1‐positive neurons of the 4HBP group relative to control (Figure S4a, Supporting Information). Similar results were also observed in NSCs isolated from the hippocampus of E14.5 mice exposed to 4HBP (Figure S4b, Supporting Information). These data suggest that 4HBP exposure affects neuronal differentiation of hippocampal NSCs.

### 2.3 Transcriptomic Analyses of Mouse NSCs Treated with 4HBP

To gain insight into the effect of 4HBP on the global gene expression of NSCs, transcriptomic analyses of mouse NSCs treated with 1 µM 4HBP under either proliferation or differentiation media were performed. Using RSEM method,^[^ ^15^ ^]^ over 19 000 genes were quantified. To evaluate the accuracy of quantification, we introduced Salmon^[^ ^16^ ^]^ for a second round of quantification. We found over 97% of genes quantified by RSEM were also quantified by Salmon, and the Pearson and Spearman correlation coefficient were over 0.84 and 0.98, respectively (Figure S5a, Supporting Information), showing the quantification by RSEM is of high accuracy. We also evaluated the gene body coverage across all transcripts in each sample and found no bias on either 5’ or 3’ end (Figure S5b, Supporting Information).

**Figure 3:**
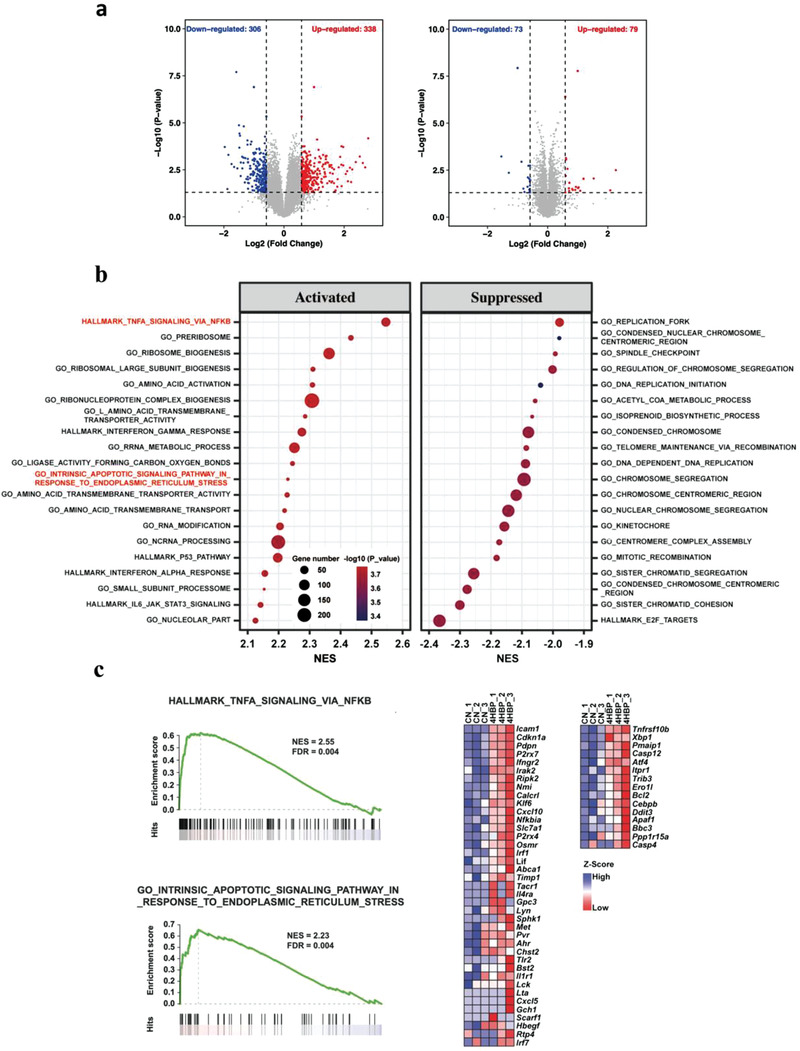
Transcriptomic analyses of mouse NSCs treated with 4HBP. a) Volcano plots of gene expression changes in 4HBP treatment versus Ctrl under proliferation and differentiation conditions, respectively. Blue dots indicate genes downregulated in 4HBP treated samples (*p* < 0.05 and log_2_FC < −0.58). Red dots indicate genes upregulated upon 4HBP treatment (*p* < 0.05 and log_2_FC > 0.58). *P*‐values were determined by the limma package. *n* = 2 for control group under differentiation condition and *n* = 3 for all the other groups. b) Gene set enrichment analysis of Hallmark and GO gene sets using pre‐ranked gene list by fold change of gene expression with 4HBP treatment under proliferation condition. The significantly enriched gene sets were identified (adjusted *p* < 0.01) and ranked by normalized enrichment score. The top 20 up‐regulated and down‐regulated gene sets are shown in the bubble plot. The selected gene sets were highlighted by red font. c) GSEA plots of selected gene sets by “draw.GSEA” function from NetBID R package (v‐2.0.2) and the heatmap of the leading‐edge genes of these gene sets by pheatmap R package (v1.0.12).

With the accurate quantification values, we proceed to differential expression analysis. The principle component analysis was introduced and showed that 4HBP treatment significantly changed the gene expression profiles under proliferation condition but not under differentiation condition (Figure S5c, Supporting Information). This is consistent with the results of differential expression analysis, where we identified 644 differential expressed genes under proliferation condition and only 152 under differentiation condition (**Figure** **3**a). These data suggest that 4HBP mainly impact on NSCs proliferation, which became the primary focus of our following investigation. The top 10 deregulated genes in proliferation condition were validated by individual qPCR (Figure S5d, Supporting Information).

Next, gene set enrichment analysis (GSEA) on hallmark and gene ontology (GO) gene sets was performed. The top 20 downregulated gene sets (*q*‐value < 0.01, ranked by NES) were associated with cell cycle related pathways (Figure 3b), corroborating that 4HBP impaired the proliferation of NSCs. To our attention, the TNF*α* signaling via NF*κ*B, inflammatory response, and the apoptotic signaling in response to ER stress were significantly enriched and highly ranked amongst the top 20 upregulated gene sets (*q*‐value < 0.01, ranked by NES), in addition to those related to ribosome biogenesis (Figure 3b,c and Figure S5e, Supporting Information). These results indicate that 4HBP may induce ER stress, inflammatory response, and apoptosis in mouse NSCs.

### 2.4 4HBP Induces PERK and NF*κ*B Signaling in Hippocampal NSCs

**Figure 4:**
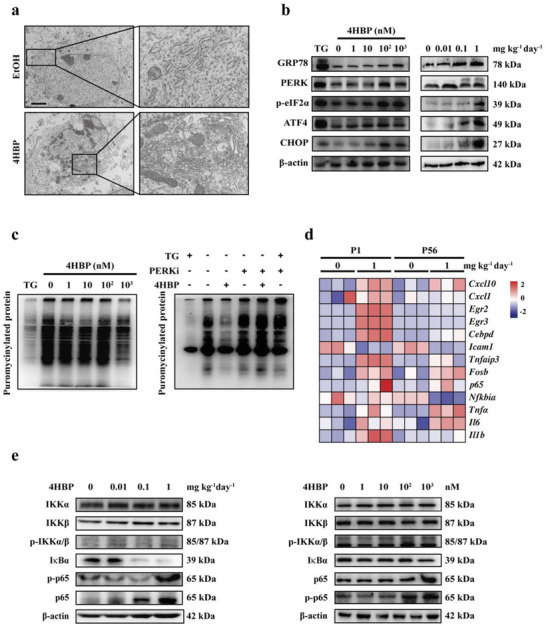
Maternal 4HBP exposure induces ER stress and inflammation in the offspring hippocampus. a) Representative TEM images of hippocampal tissues of P1 offspring mice exposed to either EtOH (control) or 1 mg kg^–1^ day^–1^ 4HBP. Magnified images of the orange box are shown to the right of each original image. Data from *n* = 3 mice per group. Scale bar, 2 µm. b, left) Western analyses of PERK pathway in hippocampal NSCs treated with different concentrations of 4HBP for 7 days in proliferation media. NSCs treated with 200 nm thapsigargin (TG) for 2 h were used as a positive control. Right, Western analyses of PERK pathway in the hippocampus of P1 offspring among different treatment groups. c, left) Primary NSCs were cultured and exposed to the indicated dose of 4HBP for 7 days, then treated with 1 µm puromycin for 1 h before being harvested in proliferation media. Right, primary NSCs were exposed to 1 µm 4HBP for 7 days and 0.5 µm GSK2606414 (PERKi) for 12 h in proliferation media before harvested. The cells were then subjected to measurement of protein synthesis by Western analyses. d) RNA was extracted from hippocampal tissues of P1 and P56 offspring exposed to indicated doses of 4HBP. The mRNA level of inflammatory cytokines was detected by qPCR. Data from *n* = 3 mice per group. e, left) Protein was extracted from hippocampal tissues of P1 offspring and subjected to Western analyses of p65 and I*κ*B*α*. Right, NSCs were treated with indicated concentrations of 4HBP for 7 days in proliferation media, then harvested for Western analyses of the NF*κ*B pathway. Results of Western blot were from a representative experiment in triplicates.

To validate that ER stress is induced by 4HBP from a morphological standpoint, we examined the ultrastructure of the hippocampal NSCs by transmission electron microscopy (TEM). A significant increase in volume and number of the ER was observed in 4HBP‐exposed NSCs both in vivo and in vitro (**Figure** **4**a and Figure S6a, Supporting Information), indicative of disrupted ER homeostasis. Since both PERK and IRE1*α* pathways are capable of inducing apoptosis,^[^ ^4^, ^17^ ^]^ we next evaluated the activation pattern of these UPR signaling in 4HBP‐treated NSCs by Western blot. The level of PERK, as well as its downstream effectors p‐eIF2*α*, ATF4, and CHOP, was concordantly activated (Figure 4b), whereas little to no change in the level of p‐IRE1*α*, p‐JNK, and ATF6 was seen (Figure S6b, Supporting Information). Furthermore, 4HBP dose‐dependently repressed protein synthesis labeled by puromycin, a phenomenon effectively reversed by addition of a PERK kinase inhibitor GSK2606414 (PERKi) (Figure 4c). In line with these findings, increased mRNA and protein expression of PERK signaling components was also observed in P1 hippocampal tissues of the 4HBP exposed offspring (Figure 4b and Figure S6c, Supporting Information). To our surprise, a mild activation of PERK pathway was still observed in hippocampus of P56 offspring (Figure S6c,d, Supporting Information), suggestive of a chronic stress response. These results suggest that 4HBP induces ER stress and activates the PERK signaling in hippocampal NSCs.

Another key indication of the transcriptomic data is the strong induction of inflammatory response, in particular the TNF*α* signaling via NF*κ*B pathway. Indeed, the mRNA expression of canonical genes involved in NF*κ*B signaling and inflammatory response, such as TNF*α*, IL‐1*β*, and IL‐6, were significantly upregulated in the hippocampal tissues of both P1 and P56 offspring (Figure 4d). Furthermore, the phosphorylated and total level of p65, the core component of the NF*κ*B transcriptional complex, was robustly increased, which was accompanied by a reduction in I*κ*B*α* level in P1 and P56 offspring (Figure 4e). Similar results were obtained in primary NSCs treated with 4HBP for 7 days, including the elevated level of p65 mRNA (Figure 4e and Figure S6f, Supporting Information). These data indicate that 4HBP triggers inflammatory response and activates the NF*κ*B signaling in hippocampal NSCs.

### 2.5 PERK‐eIF2*α* Axis Forms a Positive Feedback Loop with NF*κ*B Signaling to Promote Apoptosis in NSCs Exposed to 4HBP

The two‐sided relationship between PERK and NF*κ*B signaling has been described before,^[^ ^18^ ^]^ but their interaction in the NSCs has not been thoroughly characterized. To illustrate this, we first depleted p65 in primary NSCs by siRNA‐mediated knockdown, which resulted in significantly reduced expression of GRP78, PERK, and ATF4 in the presence of lipopolysaccharide (LPS) and 4HBP (Figure S7a, Supporting Information). These data are in keeping with the well‐established regulatory effect of NF*κ*B on PERK signaling.^[^ ^18^, ^19^ ^]^

**Figure 5:**
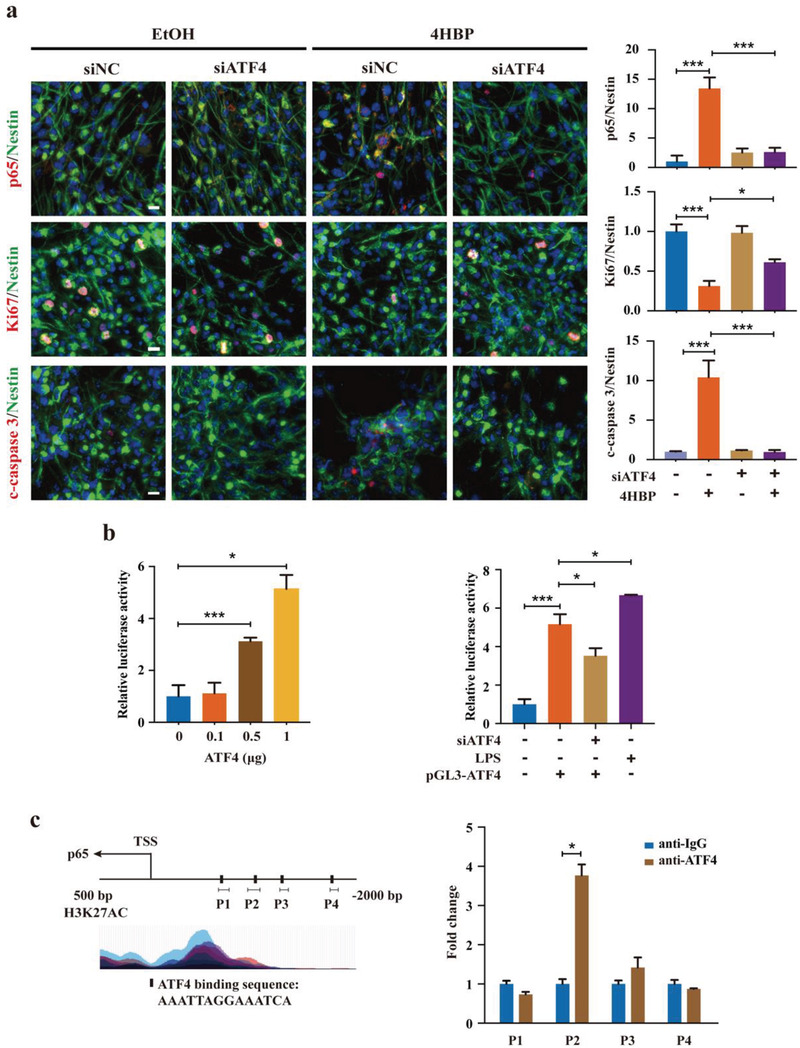
PERK pathway directly activates the NF*κ*B signaling via ATF4. a) NSCs were transfected with ATF4 siRNA followed by 1 µm 4HBP treatment for 7 days in proliferation media. Representative immunofluorescent images of co‐localization of p65, Ki67, cleaved‐caspase 3 with Nestin are shown, and the immunofluorescent signals were quantified. Scale bar, 10 µm. Three replicates per group. b, left) 293T cells were transfected with 1 µg pGL3‐p65 luciferase reporter plasmid plus either empty vector (pCDNA3) or indicated concentrations of pCDNA3‐ATF4 plasmid. Right, 293T cells were transfected with 1 µg pGL3‐p65 luciferase reporter plasmid plus empty vector (pCDNA3) or pCDNA3‐ATF4 plasmid (0.5 µg), plus the presence or absence of ATF4 siRNA. LPS (0.2 µg mL^−1^, 2 h) was used as a positive control. Luciferase activity was determined after 48 h. c, left) Prediction and validation of possible ATF4 binding sites on p65 promoter region. c, right) 293T cells were transfected with either empty pCDNA3 vector or the pCDNA3‐ATF4 plasmid. After 48 h, ChIP assay was performed using ATF4 antibody. Data are representative of two experiments in duplicates. Data are shown as the mean ± SEM. a,b, right) One‐way ANOVA with Fisher's LSD test or b, left) one‐way ANOVA with Dunnett's multiple comparisons test or c) unpaired and two‐tailed *t* test, * *p* < 0.05, ** *p* < 0.01, *** *p* < 0.001.

On the other hand, activation of PERK‐eIF2*α* has been shown to promote NF*κ*B signaling by inhibiting the translation of I*κ*B*α*, thereby leading to accumulated p65.^[^ ^20^ ^]^ Indeed, we noted a decline in the level of I*κ*B*α* upon 4HBP treatment, which was significantly reversed by GSK2606414 (PERKi) treatment (Figure S7b, Supporting Information). Despite this contributing factor to the elevated protein level of p65, it does not explain the dramatic increase in the mRNA expression of p65, which suggests that additional mechanism remains to be explored. Considering the role of ATF4 as a potent transcription factor, one possibility is that ATF4 may directly activate p65 gene expression. In support of this hypothesis, genetic inhibition of ATF4 significantly reduced the level of both total and phosphorylated p65 in the presence of 4HBP (Figure S7c, Supporting Information). Importantly, ATF4 knockdown almost completely eliminated nuclear p65 in the 4HBP‐treated NSCs, which was accompanied by increased proliferation and decreased apoptosis, as reflected by Ki67 and cleaved‐caspase 3 staining (**Figure** **5**a). Ectopic expression of ATF4 activated a p65 promoter‐driven luciferase reporter in a dose‐dependent manner in 293T cells, which was effectively attenuated by ATF4 depletion (Figure 5b). In addition, four potential ATF4 binding sites 2 kb upstream of the p65 gene transcription start site (TSS) were predicted by JASPAR database.^[^ ^21^ ^]^ Chromatin immunoprecipitation (ChIP)‐qPCR analysis confirmed that ATF4 associated with one of the predicted response elements in the promoter region of p65 gene (Figure 5c). Last, genetic ablation of either ATF4 or CHOP relieved 4HBP‐induced apoptosis in NSCs, leading to enhanced viability and improved neurosphere growth (Figure S7d,e, Supporting Information). Similar phenotype was observed upon pharmacological inhibition of PERK (Figure S7f–h, Supporting Information). Together, these results suggest that the PERK‐eIF2*α* axis forms a positive regulatory loop with NF*κ*B signaling to promote inflammatory response and apoptosis in NSCs exposed to 4HBP.

### 2.6 A PERK Inhibitor Ameliorates the 4HBP‐Induced Neurodevelopmental Toxicity in Offspring Mice

**Figure 6:**
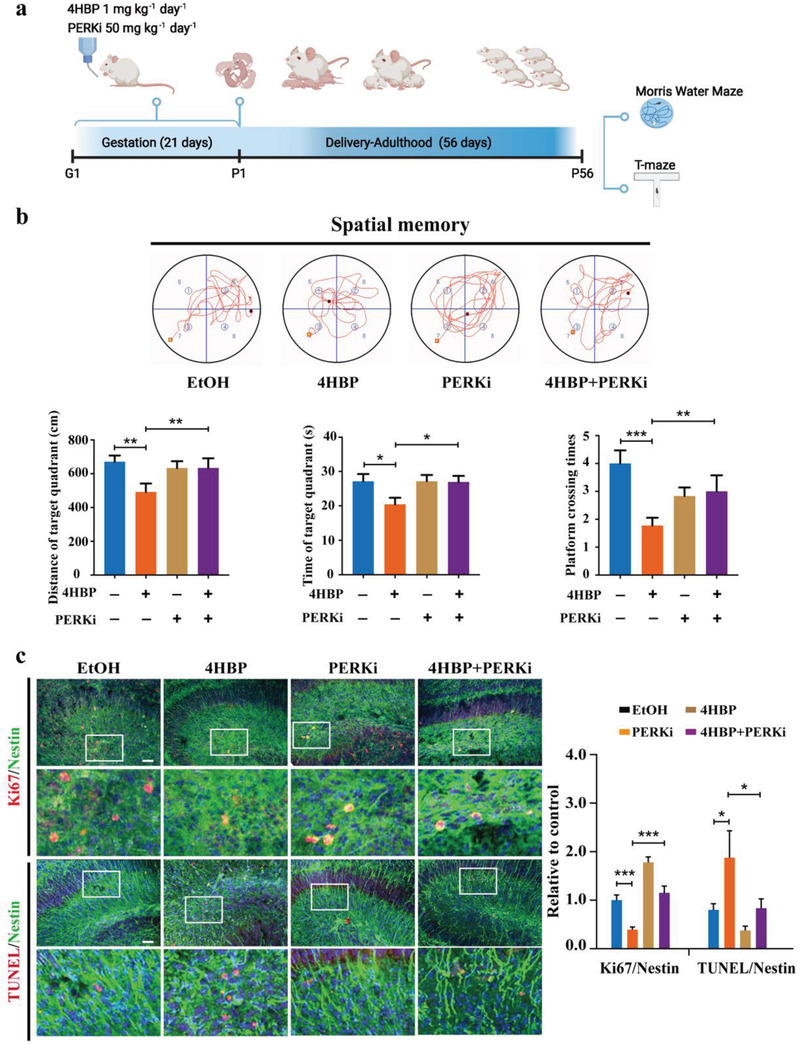
PERK blockade alleviates the toxic neurodevelopmental phenotype induced by maternal 4HBP exposure. a) Schematic overview of the experimental design. Pregnant mice were exposed to 1 mg kg^–1^ day^–1^ 4HBP via drinking water, and treated with or without 50 mg kg^–1^ day^–1^ GSK2606414 (PERKi) via oral gavage throughout the entire pregnancy. The offspring were housed under normal condition until postnatal day 56 for behavioral tests. b, top) Representative swimming trace in the spatial memory stage after the hidden platform was removed. b, bottom) Distance and time of target quadrant and platform crossing numbers in spatial memory stage among all groups (Data from *n* = 6–10 mice per group). c) Representative confocal images and quantification of NSCs co‐labeled with Nestin plus Ki67 or TUNEL in the DG region of the P1 offspring hippocampus. Scale bar, 100 µm. Data from *n* = 3 mice per group. Data are shown as the mean ± SEM. One‐way ANOVA with Fisher's LSD test, * *p* < 0.05, ** *p* < 0.01, *** *p* < 0.001. Parts of Figure 6a were made with BioRender.com.

Based on these findings, we next investigated whether pharmacological inhibition of PERK could ameliorate the 4HBP‐induced neurotoxic phenotype in offspring mice. Pregnant dams were exposed to 4HBP and treated with or without GSK2606414, and the offspring were subjected to behavioral tests (**Figure** **6**a). Strikingly, GSK2606414 of moderate dose alone did not alter the behavioral performance in the Morris water maze test, but almost completely rescued the phenotype impaired by 4HBP exposure to the control level (Figure 6b and Figure S8a, Supporting Information). Similar observation was obtained in the T‐maze test (Figure S8b, Supporting Information), verifying the improved cognitive function of offspring mice by PERK inhibition. Consistently, enhanced proliferation and weakened apoptosis were observed in the NSCs in the hippocampus of P1 and P56 offspring exposed to 4HBP (Figure 6c and Figure S8c, Supporting Information). Moreover, 4HBP‐induced PERK‐eIF2*α* pathway activation, p65 accumulation, activated NF*κ*B signaling, as well as elevated level of inflammatory cytokines were ameliorated by GSK2606414 in hippocampus of offspring (Figure S8d,e, Supporting Information). These results suggest that inhibition of PERK alleviates the 4HBP‐induced apoptosis of NSCs in offspring mice, leading to improved hippocampus development and cognitive function.

### 2.7 PERK Blockade Ameliorates the 4HBP‐Induced Neurotoxicity in Human Brain Organoids

**Figure 7:**
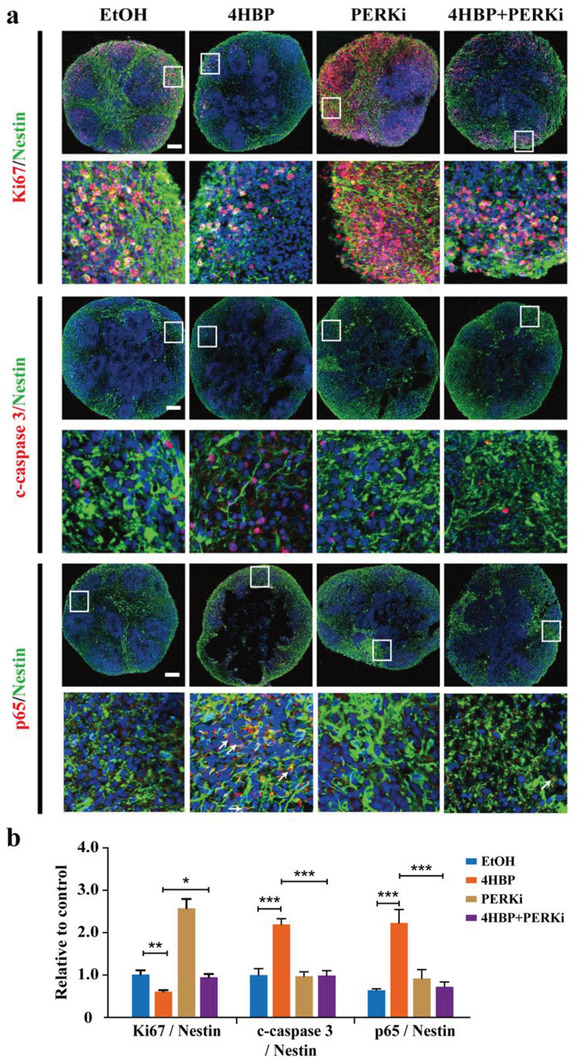
PERK inhibition alleviates 4HBP‐induced apoptosis and p65 activation in human brain organoids. Human brain organoids were exposed to 2 weeks of 0.5 µm 4HBP, and supplemented with or without 0.1 µm GSK2606414 (PERKi) for 1 week, then cultured in normal media and allowed for organoid growth for 2 months. a) Representative confocal images of NSCs co‐labeled with Nestin and Ki67 or cleaved‐caspase 3 in human brain organoids. Magnified images of the white box are shown underneath each original image. Scale bar, 100 µm. b) Quantification of immunofluorescent images in (a). Data from *n* = 3 brain organoids per group. Data are shown as mean ± SEM. One‐way ANOVA with Fisher's LSD test, * *p* < 0.05, ** *p* < 0.01, *** *p* < 0.001.

Last, to explore the translational significance of our findings, we utilized a human brain organoid model derived from the induced pluripotent stem cells (iPSCs). The human NSCs were exposed to 2 weeks of 4HBP, and supplemented with or without GSK2606414 for 1 week, then cultured in normal media and allowed for organoid growth for 2 months (Figure S9a, Supporting Information). There was no significant difference in the size and morphology of organoids among different treatment groups (Figure S9b, Supporting Information). Interestingly, histological examination showed that the organoids exposed to 4HBP displayed marked necrosis in the internal area, which was largely reduced by GSK2606414 (Figure S9c, Supporting Information). Mirroring the observations in mice, 4HBP inhibited proliferation as reflected by Ki67 staining, whereas strongly triggered apoptosis was represented by cleaved‐caspase 3 staining. Furthermore, 4HBP robustly enhanced the intensity of p65 staining, in particular nuclear p65 signals. Importantly, these adverse phenotypes were completely reversed in the organoids supplemented with GSK2606414 (**Figure** **7**a,b). These results imply that repression of PERK signaling attenuates the 4HBP‐induced neurotoxicity in human brain organoids.

## 3 Discussion

Despite the widespread use and exposure of BPs, our knowledge on their neurodevelopmental toxicity is scarce. The significant association between maternal exposure to 4HBP and child cognitive development in our previous epidemiological study prompts us to determine their causal relationship and the underlying molecular mechanism. In this study, we expose pregnant mice to environmentally relevant dose of 4HBP that mimics the human exposure condition, which leads to significantly impaired hippocampal development and cognitive function in offspring. Meanwhile, 4HBP represses in vitro proliferation and differentiation of mouse NSCs, and retards the development of human iPSCs‐derived neural organoid growth. Together, these results record the previously underrated neurodevelopmental toxicity of 4HBP. Interestingly, the parental BP fails to affect the in vitro well‐being of NSCs at the same dosage as 4HBP does, suggesting that the neurotoxicity is significantly augmented when BP is metabolized into 4HBP. In addition, BP‐3 displayed little toxicity on NSCs in vitro, while maternal levels of BP‐3 and BP‐1 seem not associated with child neurodevelopmental abnormality in our previous cohort study either. However, this does not rule out the possible toxicity of other BP derivatives, which warrants further characterization.

Functional analysis and transcriptomic profiling reveal that 4HBP mainly affects proliferation, instead of differentiation, of NSCs, by inducing inflammatory response and apoptosis. Molecular dissection further uncovers that PERK branch of the UPR forms a positive feedback loop with NF*κ*B signaling, which plays a central role in mediating the 4HBP‐induced neurotoxicity. Importantly, blockade of PERK kinase activity ameliorates the 4HBP‐triggered neurodevelopmental toxicity and cognitive dysfunction in offspring mice, a phenotype that is recapitulated in a human brain organoid model. Dysregulated translation has been previously implicated in neurodevelopmental disorders.^[^ ^22^ ^]^ Furthermore, a recent study reports that intrauterine inflammation evokes the eIF2*α*‐driven integrated stress response and disrupts fetal brain development.^[^ ^3c^ ^]^ In line with these observations, our findings affirm that proteostatic stress and inflammatory response may be common and concomitant features of neurodevelopmental toxicity in response to environmental influence.

**Figure 8:**
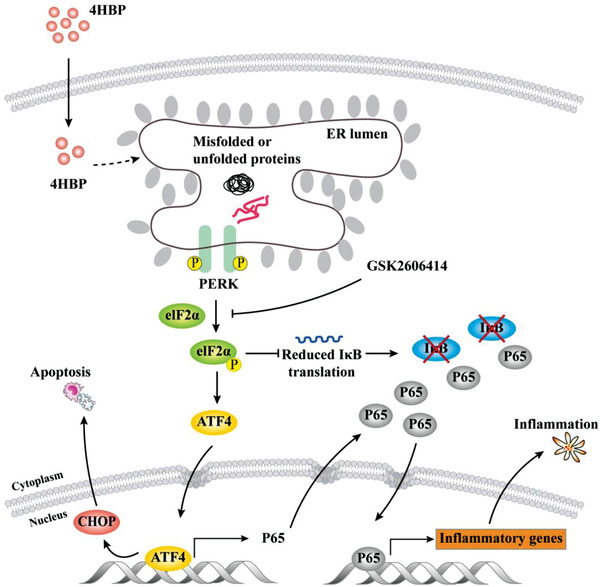
Schematic summary of the present study. Maternal exposure to 4HBP induces ER stress, inflammation, and apoptosis in the offspring hippocampal NSCs, thereby causing neurodevelopmental disorders. This is achieved by the interplay between the PERK‐eIF2*α* branch of the UPR and NF*κ*B signaling. PERK activation induces CHOP and triggers apoptosis, whereas it also promotes NF*κ*B signaling by translational inhibition of I*κ*B*α* via eIF2*α* phosphorylation, and transactivation of p65 via ATF4. PERK blockade disrupts this signaling loop and significantly improves the 4HBP‐induced neurodevelopmental toxicity.

The bidirectional interplay between PERK and NF*κ*B signaling has been illustrated before.^[^ ^18^ ^]^ In this specific context, we unveil a positive feedback regulatory relation between these pathways, as perturbation of either signaling impedes the activity of the other. Importantly, we show that the PERK downstream effector ATF4 transcriptionally activates p65 expression. Inhibition of either PERK activity or ATF4 results in drastically decreased p65 total level, nuclear translocation, and signaling activity. Therefore, our results provide a novel and direct mechanistic connection within this molecular circuit, and suggest that inhibition of PERK kinase activity represses NF*κ*B signaling on multiple levels (**Figure** **8**).

In conclusion, our study cautions that application of BP‐supplemented personal care products during pregnancy may lead to offspring neurodevelopmental toxicity, which can be ameliorated by inhibition of PERK signaling activity.

## 4 Experimental Section

### Animal Experiments

Male (25–30 g) and female (20–25 g) C57BL/6 mice were purchased from experimental animal research center of Shanghai JSJ. The animal experimental procedure was strictly conducted according to guidelines and approved by the Institutional Animal Care and Use Committee of Huazhong University of Science and Technology. Mice were placed in a room at the temperature of 20–25 °C, the humidity of 40–50%, and under a 12‐h light/dark cycle. Adult female mice were mated with male mice at 2:1. Pregnancy was determined by the presence of the vaginal plug. Pregnant mice were exposed to 0.1, 1 mg kg^–1^ day^–1^ 4HBP and EtOH (as vehicle control) via oral delivery. The PERK kinase inhibitor GSK2606414 (50 mg kg^–1^ day^–1^) was treated by oral gavage. Newborn offspring were sacrificed within 24 h after delivery and hippocampal tissues were collected. The rest of the offspring were raised until postnatal day 56 for behavioral tests.

### Morris Water Maze Test

Morris water maze test was performed according to the nature protocols.^[^ ^23^ ^]^ The experiments included 2 different assessments: spatial learning test and spatial memory test. All tests were performed in a quiet room with indirect lighting. The device had a swimming pool of 170 cm in diameter, and the water temperature was at ≈20–22 °C. The swimming pool was virtually divided into four equal quadrants. The white edible pigment was used to opaque water that helps to hide the underwater platform. The hidden circular platform of 9 cm in diameter located in the quadrant of northeast (upper right) and submerged 1.5 cm below the surface of the water and remained constant during the entire spatial learning test. The entire experiment lasted 5 or 6 days, of which the spatial learning test lasted 4 or 5 days. Each trial had a maximum of 1 min with an inter‐trial interval of 15 s. Each animal was tested four times a day. The animal was placed in a specific starting location in the maze, as described in the article.^[^ ^23^ ^]^ If animals failed to reach the platform within 1 min, they were guided to the platform using a guide stick. After animals reached the escape platform, they were allowed to remain there for 15 s before the next trial. The escape latency was defined as the time that animals spent in swimming from the starting position to the platform. The spatial memory test was performed on the last day. In this procedure, the platform was removed and the animals were allowed to swim for 60 s. The whole experiment was monitored by a camera above the center of the pool.

### Step‐Through Test

The apparatus was divided into dark and transparent rooms. For training, the mice were placed in the transparent compartment and allowed to enter the dark compartment through the door in between. Immediately after entry, a scrambled foot shock (36 V, 55 Hz) was delivered to the mice, which were allowed to escape to the safe transparent compartment. Each mouse was given a maximum of 5 min to train. For animals failed to enter the dark compartment within 5 min, they were guided to the dark compartment using a guide stick. 24 h after training, the mice were again placed in the safe transparent compartment. The response latency to enter the dark compartment and the times of entering into the dark compartment in 5 min were measured.

### T‐Maze Test

T‐maze alternation has been used for decades in academia and industry for its sensitivity in assessing cognitive dysfunction and its simplicity of construction. Enclosed T‐maze, an apparatus with 10 cm‐wide floor and 20 cm‐high walls in the form of a “T” placed horizontally, was used. The stem of the two goal arms and a start arm were all 30 cm long and every arm had a guillotine door. First, all the doors were open and the mouse directly from its home cage, was placed in the start area and allowed to select the left or right arm. The mouse was kept in the chosen arm by quietly sliding the door down. After 30 s, the mouse and central partition were removed, and the mouse was returned to its holding cage. After a retention interval of 1 min, the mouse was placed in the start area for a second trial with all the doors open. Spontaneous exploration was repeated 15 times as described above. Each exploration should take no more than 2 min. If one mouse fails to run within 90 s, a reasonable criterion at which to abort the trial, it was removed and tested again after resting. The percentage of spontaneous exploration was analyzed.

### Cell Culture

Primary NSCs were obtained from embryonic hippocampus on embryonic day 14.5. Briefly, the extracted tissue was digested for 10 min by trypsin and then gently dispersed. The suspension was filtered through a 40 mm filter and then collected. The hippocampus‐derived NSCs were cultured in suspension and maintained proliferation in DMEM/F12 (#11330‐032, Gibco) containing 20 ng mL^−1^ EGF (#AF‐450‐33, Peprotech), 20 ng mL^−1^ bFGF (#315‐09, Peprotech), 1% glutaMAX (Gibco), 1% B27(#17504‐044, Gibco) and 1% penicillin/streptomycin (Meilunbio). After 4–5 days, neurospheres occurred and were dissociated into single cells by accutase (#A11105‐01, Gibco) and then plated on plates coated with poly‐l‐ornithine (Sigma) and laminin (L2020, Sigma) in growth media.

### Neurosphere Growth Kinetics Assay

Hippocampus‐derived primary NSCs were passaged by gentle digestion. Then, the single‐cell suspension of NSCs was plated in a 6‐well plate at a density of 50 000 cells per well and allowed for growth for 7 days. The diameter of neurospheres was analyzed on day 7.

### Cell Viability Assay

NSCs were plated in 96‐well plates, treated with the indicated drug. 7 days later, cell viability was determined using the Cell Count Kit‐8 (#A311‐01, Vazyme) according to the manufacturer's instructions. Absorbance was measured at 450 nm using a microplate reader (Thermo Fisher Scientific).

### Immunofluorescence

NSCs were fixed with 4% paraformaldehyde (PFA) (Servicebio) for 10 min and then permeabilized with 0.3% Triton X‐100 for 1 h. Subsequently, NSCs were blocked with 5% BSA and incubated with primary antibodies at 4 °C overnight. This was followed by incubation of fluorochrome‐conjugated secondary antibodies at room temperature for 1 h. The primary antibodies Nestin (ab22035, 1:500), Ki67 (ab15580, 1:500) were from Abcam; Nestin (#4760, 1:500), cleaved‐caspase 3 (#9664, 1:500), Tuj1 (#4466, 1:500), p65 (#8242, 1:500) were from Cell Signaling Technology, NeuN (# 26975‐1‐AP, 1:200) was from Proteintech and GFAP (PB9082, 1:500) was from Boster. Donkey anti‐rabbit alex fluor 594 (#711‐585‐152, 1:1000) and donkey anti‐mouse alex fluor 488 (#715‐545‐150, 1:1000) were from Jackson.

For hippocampal tissue section staining, mice were anesthetized with chloral hydrate, and the brains were collected and fixed with 4% PFA and dehydrated with 30% sucrose solutions. Brain tissues were sectioned at 30 µm with a cryostat (Leica Biosystems). Brain sections were blocked and permeabilized with 5% BSA/0.3% Triton X‐100/PBS. The subsequent staining procedure was the same as described above. Images were acquired using the Flu View FV1200 confocal microscope (Olympus) and processed using Photoshop. Quantification of the positive staining signals was performed in three representative brain sections for each mouse.

### TUNEL Staining

Cells or tissues were fixed with 4% PFA, washed with PBS, and the TUNEL assay was performed according to the manufacturer's protocol (Meilunbio).

### Western Blot

Total proteins were extracted from hippocampus tissues or NSCs using RIPA lysis buffer (Beyotime) supplemented with phosphatase and protease inhibitors and PMSF, and then quantified by BCA assay (Meilunbio). Western blot was performed as previously described.^[^ ^24^ ^]^ GRP78 (#3711; 1:1000), PERK (#3192; 1:1000), p‐eIF2*α* (#9721; 1:1000), CHOP (#2895; 1:1000), IRE1*α* (#3294; 1:1000), p‐IRE1*α* (#9721S, 1:1000) and NF‐*κ*B pathway sampler kit (#9936) were from Cell Signaling Technology; ATF6 (#37149, 1:1000) was from Abcam. *β*‐actin (MA5‐15739, 1:5000) was from Invitrogen; GAPDH (#60004, 1:5000) was from Proteintech. The horseradish peroxidase‐conjugated anti‐rabbit IgG (SA00013‐3, Proteintech) or anti‐mouse IgG (SA00001‐2, Proteintech) secondary antibodies were used, followed by detection with the enhanced chemiluminescence system (GE Healthcare). ECL Western blot analysis system was utilized for detection of the immunoreactive bands according to the manufacturer's instructions by using the GeneGnome system (Syngene). *β*‐actin or GAPDH was used as a loading control.

### RNA Extraction and Real‐Time qPCR

Total RNA from NSCs or hippocampus was extracted by TRIzol reagent box (#15596018, Thermo Fisher Scientific). Single‐strand cDNA was synthesized using a universal cDNA synthesis kit (#R233‐01, Vazyme). The expression of mRNA was tested by a fast real‐time qPCR system (7900 HT, ABI, Thermo Fisher Scientific) using a SYBR Green master mix (#Q311‐02, Vazyme). Primer sequences are listed in Table S1, Supporting Information. The mRNA level was normalized against that of *β*‐actin or GAPDH.

### TEM

The NSCs were collected by centrifugation at 2500 rpm for 5 min. Hippocampal tissues were sliced into pieces of 1 mm^3^. The samples were immersed in 2.5% glutaraldehyde immediately after being isolated from the brain at 4 °C for 6 h. Then the pieces were fixed with 1% osmium tetroxide and dehydrated in graded ethanol series, and embedded in Araldite. Ultrathin sections (50 nm) were stained with 2% uranyl acetate for 15 min and 2% lead citrate for 15 min. Finally, the sections were observed under a TEM (Philips Tecnai 10). Three replicates were included for each treatment group.

### Luciferase Reporter Assay

293T cells were cultured in six‐well plates, transfected with ATF4 siRNA, p65 luciferase reporter plasmid, empty vector (pCDNA3), and pCDNA3‐ATF4 plasmid of the indicated concentration for 48 h before harvest. Luciferase activity was assayed using the Firefly & Renilla Luciferase Reporter Assay Kit (#MA0518, Meilunbio).

### ChIP

Potential ATF4 binding sites 2 kb upstream of the P65 gene TSS were predicted by JASPAR database (http://jaspar.genereg.net/). Under the given settings, four overlapping binding regions were identified and corresponding primers (Table S1, Supporting Information) were then designed and used for ChIP‐qPCR experiment. ChIP‐qPCR experiment was performed using a CHIP Assay Kit (#P2078, Beyotime). Briefly, crosslinking was performed with 1% formaldehyde at 37 °C for 10 min and then quenched with 125 mm glycine. The cells were lysed in SDS buffer and sonication (VXC‐750, Sonics) was used to fragment the DNA in 200–1000 bp. After sonication, the samples were centrifuged at 12 000 g at 4 ℃ for 5 min and supernatant was collected. Sheared chromatin was immunoprecipitated overnight with an ATF4 antibody (11815s, Cell Signaling Technology), or mouse IgG. Antibody bound chromatin complexes were then immunoprecipitated with protein A‐agarose beads, and washed the precipitate with the appropriate solution. The precipitation was then dissolved in elution buffer. The cross‐links between genomic DNA and proteins were unraveled at 65 °C overnight and then the purification of DNA was performed. Immunoprecipitated DNA, as well as input DNA, was quantified by qPCR using specific primer sets as indicated.

### The Human Brain Organoid

Human iPSCs DYR0100 were purchased from Stem Cell Bank of Chinese Academy of Science. In order to culture cells in a 3D system, human iPSCs were embedded into an extracellular matrix (ECM), subsequently cultured in spinning bioreactors to promote tissue expansion and neural differentiation. Neural precursor cells were differentiated from DYR0100 for 60 days. The 3D brain organoids were generated, treated with the indicated dose of 4HBP, and cultured in human 3D cerebral organoid medium (DMEM/F12 medium (Gibco) supplemented with 1% B27 (Gibco)) for 60 days. For immunofluorescence, the 3D brain organoids were fixed by 4% PFA at room temperature for 3 h and dehydrated at 4 °C by 30% sucrose solution and embedded in Richard‐Allan Scientific Neg‐50 (Thermo Fisher Scientific). The frozen tissues were sectioned at 20 µm thickness before being subjected to immunofluorescent staining.

### Transfection of siRNA

The siRNAs were transfected with Lipofectamine RNAiMAX in Opti‐MEM (Thermo Fisher Scientific) following the manufacturer's protocol. The siRNA sequences are as follows: ATF4: 5’‐AUC GAA GUC AAA CUC UUU CUU‐3’; CHOP: 5’UGU UUC CGU UUC CUA GUU CUU‐3’ and negative control siRNA (siNC): 5’‐UAA UGA AUU GGA ACG CAU A TT‐3’. These sequences were purchased from GenScript.

### Measurement of Protein Synthesis with Surface Sensing of Translation

Surface sensing of translation method was conducted as previously described.^[^ ^25^ ^]^ Briefly, NSCs were treated with 4HBP or vehicle (ethanol) for 7 days. 1 µm puromycin (Sigma) was then added to the cultures, which were incubated for an additional 1 h before the cells were harvested. Then, proteins were extracted, quantified, resolved by SDS‐PAGE, and transferred to PVDF membrane. The blotted membrane was incubated with a primary antibody against puromycin (MABE343, 1:5000), followed by an HRP‐conjugated secondary antibody.

### Exposure Assessment of 4HBP in Mouse Tissues

50 g of tissue samples were added with 100 µL of distilled water and homogenized by ultrasonic for 30 min, then added 400 µL of methanol and vortex mixed for 3–5 min. The suspension was centrifuged for precipitation at 13 000 rpm. 450 µL supernatant was collected and concentrated into dry powder. 50 µL of methanol was then added to the powder and grinded by ultrasonic for 30 min. Samples were vortex mixed and centrifuged 10 min at 13 000 rpm and 30 µL of the supernatants were collected and taken into the injection vial to detect 4HBP. Serum was directly added with methanol and the subsequent procedure was the same as described above. The tissue concentration of 4HBP was detected by an Agilent high‐performance liquid chromatography 1260 coupled to an Agilent model 6460 tandem mass spectrometer with electrospray ionization (HPLC‐MS/MS‐ ESI).

### RNA Extraction, Sequencing, and Data Analysis

The Hippocampus‐derived primary NSCs were cultured under either proliferation or differentiation conditions. In each condition, the cells were treated by either EtOH or 1 µm 4HBP for 7 days. Total RNA was extracted using TRIzol reagent (Invitrogen). The RNA sequencing was performed at Shanghai OE Biotech, where the TruSeq Stranded mRNA LTSample Prep Kit (Illumina) was used for library generation, and 150 bp paired end sequencing was performed using an Illumina HiSeq X Ten instrument (Illumina). All relevant sequencing data are available at GEO (GSE166506).

The adapters used in library preparation were identified by FastQC (v‐0.11.5) (https://www.bioinformatics.babraham.ac.uk/projects/fastqc/) and trimmed from the raw reads by cutadapt (v‐1.13) (https://doi.org/10.14806/ej.17.1.200) using the default parameters. RSEM (v1.3.0),^[^ ^26^ ^]^ coupled with Bowtie2 (v2.2.9),^[^ ^27^ ^]^ were used to quantify the expression of genes and transcripts based on the reference genome mm10 (GRCm38) with gene annotation from GENCODE (release vM17). Raw gene‐level counts were normalized to counts per million (CPM) and further transformed by log2(CPM+1). The principal component analysis (PCA) was performed by NetBID (v‐2.0.2)^[^ ^28^ ^]^ to assess the overall similarity between samples. The differential expression analysis was conducted using limma R package (v‐3.42.2).^[^ ^29^ ^]^ The GSEA was performed by the fgsea R package (v1.12.0) (https://doi.org/10.1101/060012) with MSigDB dataset (v‐6.1)^[^ ^30^ ^]^ and visualized by “draw.GSEA” function from NetBID software (v‐2.0.2).^[^ ^28^ ^]^

### Morphological Analysis of Tuj1‐Positive Cells

The morphological complexity of Tuj1‐positive cells was performed as previously described.^[^ ^31^ ^]^ Briefly, 8‐bit images of cultured neurons were traced using the NeuronJ software (USA) and tracing files were generated. 20 mm‐spaced concentric circles were superimposed onto the image of each selected neuron and centered on its soma. For each neuron, 1) the total number of times neurites intersected any given circle (sum of intersections); 2) the number of terminal branchings; and 3) the total length of neuronal dendrite were recorded. Neurons from three independent experiments were analyzed per each experimental condition.

### Histological Examination of Hippocampus

Three 30 µm sagittal sections were sampled every 180 µm of the hippocampus per animals, followed by HE staining according to the manufacturer's protocol (Meilunbio). The slides were imaged on the Olympus DP70 macroscope. As previously described,^[^ ^32^ ^]^ hippocampal cross‐sectional area on each section was measured by tracing using ImageJ and multiplied by 360 µm (distance between sections) to estimate hippocampus volume. The estimated volume ratio of the hippocampus was calculated and analyzed using Prism v.7 software (GraphPad Software).

### Statistical Analysis

Data were shown as mean ± SEM. The unpaired and two‐tailed *t* test was employed for the comparisons between two groups, while analysis of one‐way ANOVA with Dunnett's test for comparing multiple experimental groups with a single control group and one‐way ANOVA with Fisher's LSD test for comparing among three or more groups. All the comparisons were conducted by Prism v.7 software (GraphPad Software).

## Conflict of Interest

The authors declare no conflict of interest.

## Author Contributions

F.C. and Q.P. contributed equally to this work. F.C., S.X., Y.T., and X.S. designed the study. F.C., S.W., and Y.S. performed in vitro experiments. F.C., R.W., and T.Z. performed animal experiments. Q.P. and F.Z. performed bioinformatics analysis. J.H., H.Z., Q.W., and Y.J. provided reagents. W.X., Y.L., G.‐Y.Y., W.D.V., and J.‐P.T. provided guidance on the design of experiments and interpretation of results. F.C., Q.P., and X.S. wrote the manuscript. S.X., Y.T., and X.S. edited the manuscript.

## Supporting information

Supporting Information

## Data Availability

The RNA‐Seq data described in this study are openly available in Gene Expression Omnibus under the accession number of GSE166506 (URL: https://www.ncbi.nlm.nih.gov/geo/query/acc.cgi?acc=GSE166506).
