# Supplementary material for: Maternal Benzophenone Exposure Impairs Hippocampus Development and Cognitive Function in Mouse Offspring
Source: Adv Sci (Weinh). 2021 Oct 28;8(23):2102686. doi: 10.1002/advs.202102686 (PMC8655188; doi:10.1002/advs.202102686)
Supplement: Supplementary file 1 — Supporting Information [file ADVS-8-2102686-s001.pdf]

© 2021 Wiley-VCH GmbH

ADVANCED SCIENCE
Open Access

Supporting Information

for *Adv. Sci.,* DOI: 10.1002/advs.202102686

Maternal benzophenone exposure impairs hippocampus development and cognitive function in mouse offspring

Fengzhen Cui, Qingfei Pan, Siyi Wang, Faming Zhao, Runxin Wang, Tingting Zhang, Yaying Song, Jun He, Haolin Zhang, Qiang Weng, Yang Jin, Wei Xia, Yuanyuan Li, Guo-Yuan Yang, Winnok De Vos, Jean-Pierre Timmermans, Shunqing Xu*, Yaohui Tang*, Xia Sheng*

Supporting Information

Maternal benzophenone exposure impairs hippocampus development and cognitive function in mouse offspring

Fengzhen Cui[1#], Qingfei Pan[2#], Siyi Wang[3], Faming Zhao[1], Runxin Wang[1], Tingting Zhang[1], Yaying Song[4], Jun He[5], Haolin Zhang[6], Qiang Weng[6], Yang Jin[7], Wei Xia[1], Yuanyuan Li[1], Guo-Yuan Yang[4], Winnok De Vos[8], Jean-Pierre Timmermans[8], Shunqing Xu[1*], Yaohui Tang[4*], Xia Sheng[1*]

Supplementary Figure 1

a

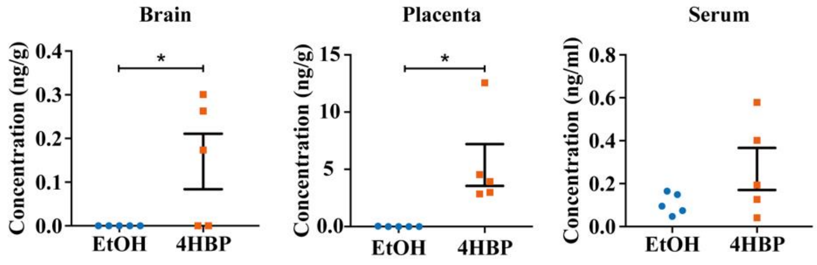

Sample	This study: Concentration (mean ± SD, n=5)	Human studies: Concentration (median (5th - 95th))	Reference
Newborn brain	0.1473 ± 0.06358 ng g^{-1}	-	-
Maternal serum	0.2683 ± 0.09777 ng ml^{-1}	ND	11b
		0.6 - 1 ng ml^{-1}	13a
Placenta	5.379 ± 1.822 ng g^{-1}	ND -1.2 ng g^{-1}	10b
		0.19 (0.20 - 2.72) ng g^{-1}	13b
		0.70 - 1.33 ng g^{-1}	13c

b

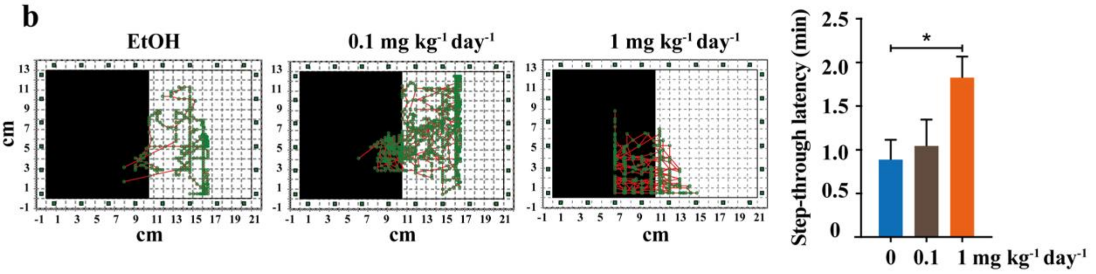

Supplementary Figure 1. Comparison of internal exposure level between our mice study and previous human studies. a, Top, pregnant mice were exposed to 1 mg kg^{-1} day^{-1} 4HBP for 20 days and the level of 4HBP in the brain, placenta and serum were assessed by HPLC-MS (Data from n = 5 mice per group). Bottom, Comparison between the level of 4HBP in our study with that in different human cohorts previously reported by us and others. **b**, Representative trace in smart cage and time spent in the dark box (Data from n = 10 mice per group). Data are shown as mean ± SEM. Unpaired and two-tailed t test (**a**) or one-way ANOVA with Dunnett's multiple comparisons test (**b**), * $p < 0.05$.

Supplementary Figure 2

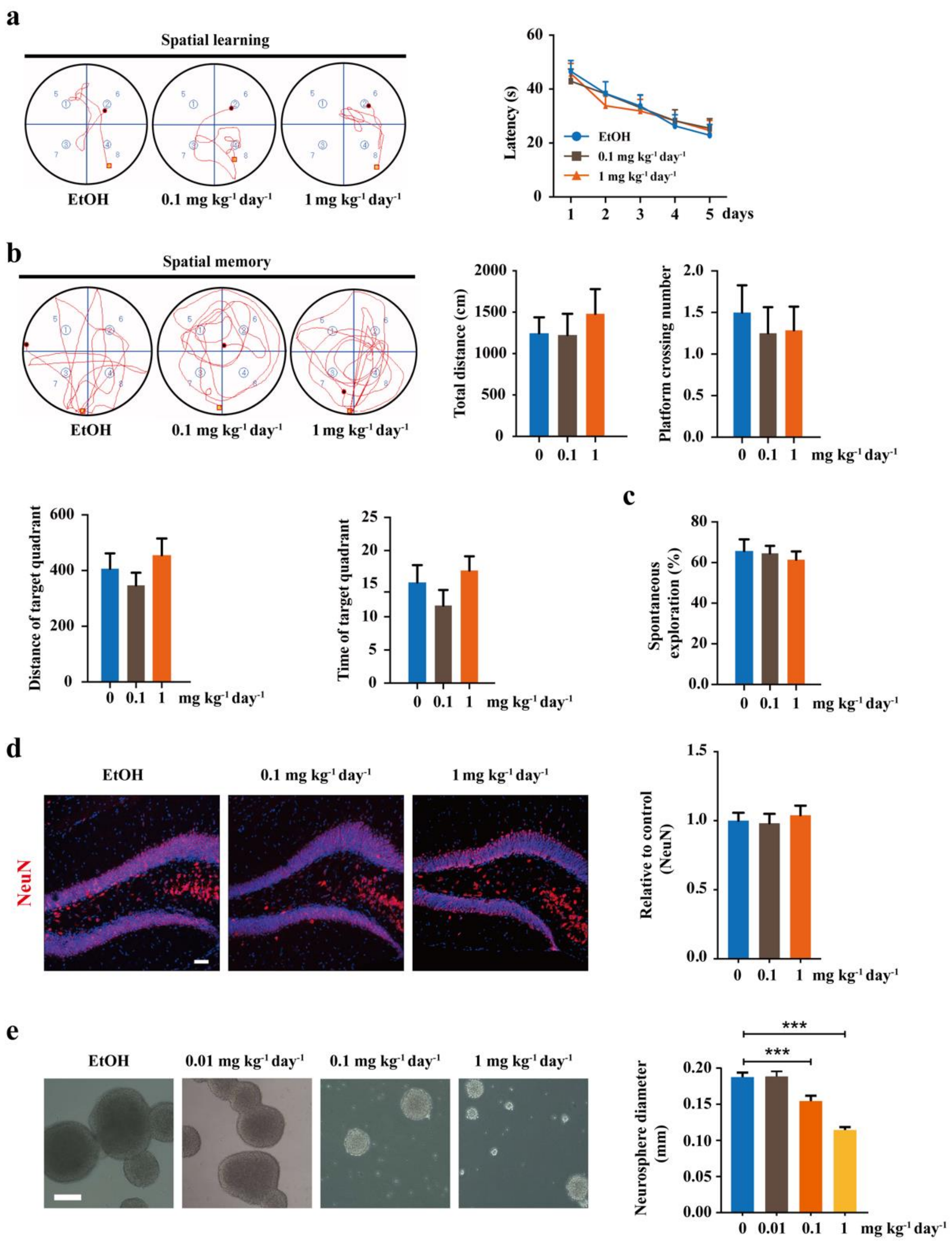

Supplementary Figure 2. 4HBP exposure of the same dosage and duration fails to induce memory and learning impairment in adult mice. Adult mice (eight-week-old) were exposed to either vehicle (EtOH) or 4HBP (0.1 or 1 mg kg^{-1} day^{-1}) via drinking water for 20 days under normal condition before performing

behavior tests. **a**, Left, representative swimming trace of mice from the start position to the platform in the spatial learning test on day 5. On the right, line graphs showed the change in escape latency from day 1 to day 5 in the spatial learning test (Data from n = 6 mice per group). **b**, Representative swimming trace in the spatial memory test after the hidden platform was removed on day 6. Bar graphs showed the total distance, times of mice crossing the platform, distance and time spent in the target quadrant in spatial memory test among different groups (Data from n = 6 mice per group). **c**, Quantification of spontaneous exploration behaviors of mice in the T-maze. Data from n = 8 mice per group (Data from n = 6 mice per group). **d**, Left, representative confocal images of NeuN in the dentate gyrus (DG) region of the hippocampus on adult mice. Scale bar, 50 μm. Right, Quantification of immunofluorescent staining of NeuN. Data from n = 3 mice per group. **e**, NSCs were derived from hippocampal NSCs isolated from offspring of the indicated treatment and cultured for 7 days in proliferation media. The neurospheres were imaged and the diameter was quantified (n = 150 – 300 neurospheres in each group). Scale bar, 100 μm. Data are shown as mean ± SEM. One-way ANOVA with Dunnett's multiple comparisons test, *** $p < 0.001$.

Supplementary Figure 3

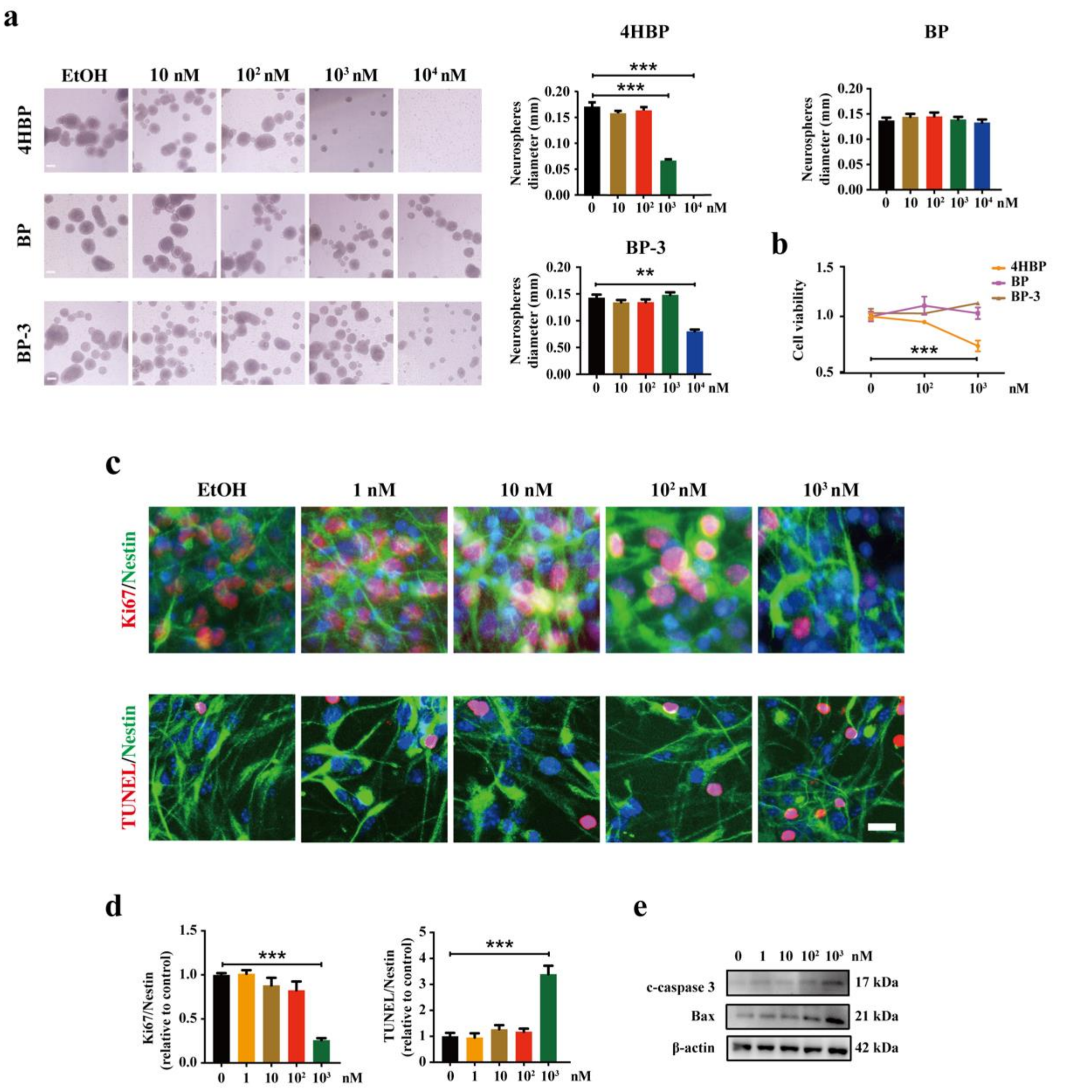

Supplementary Figure 3. 4HBP inhibits proliferation and induces apoptosis of hippocampal NSCs *in vitro*. a, Primary hippocampal NSCs were seeded into 96-well plates and treated with the indicated concentrations of 4HBP, BP and BP-3 for 7 days in proliferation media. NSCs were cultured as suspension to allow for neurosphere formation. Neurosphere diameters were quantified on day 7 (n = 150 – 300 neurospheres in each group). Scale bar, 100 μm. **b,** As in **a**, but CCK-8 assay was performed to test cell viability. Five replicates per group. **c**, Primary hippocampal

NSCs were seeded into 24-well plates and treated with the indicated concentrations of 4HBP for 7 days in proliferation media. The cells were then co-labeled with Nestin and Ki67 or TUNEL. Scale bar: 25 μm. **d**, Quantification of the immunofluorescent signals in **c**. **e,** As in **c,** NSCs were harvested and subjected to Western analysis. Results were from a representative experiment in triplicates. Data are shown as mean ± SEM. One-way ANOVA with Dunnett's multiple comparisons test, ** $p < 0.01$, *** $p < 0.001$.

Supplementary Figure 4

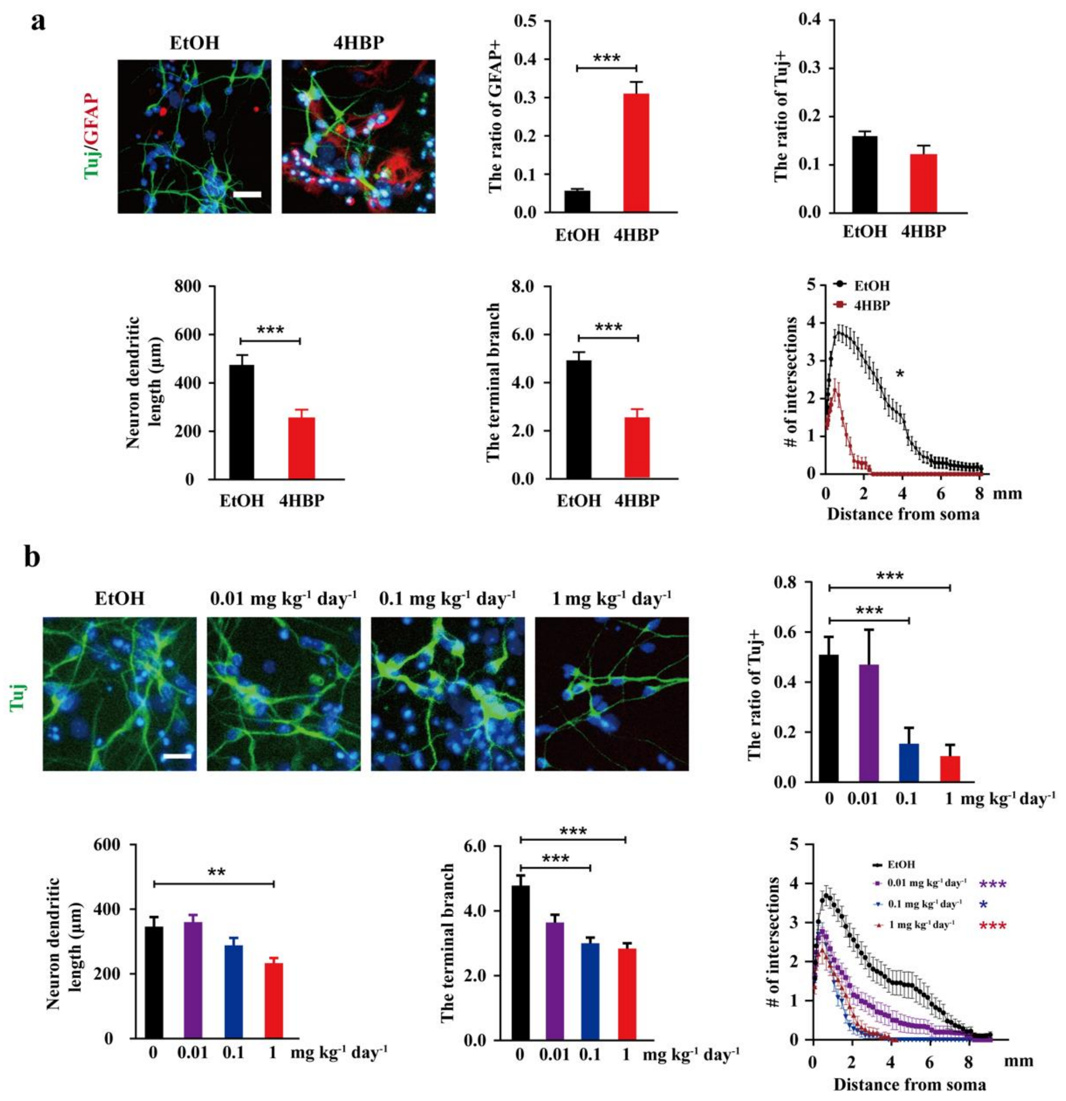

Supplementary Figure 4. 4HBP suppresses differentiation of primary hippocampal NSCs. a, Primary hippocampal NSCs were cultured and treated with 1 μM 4HBP for 7 days in differentiation media. Representative images of immunofluorescence of Tuj1 and GFAP. Scale bars, 25 μm. Analysis of the differentiation of NSCs and morphological complexity of Tuj1-positive cells. 26 – 50 neurons were analyzed. **b**, NSCs were derived from hippocampal NSCs isolated from offspring of the indicated treatment and cultured for 7 days in differentiation media.

Representative images of immunofluorescence of Tuj1 at 7 days. Scale bars, 25 μm. Analysis of NSCs differentiation of and morphological complexity of Tuj1-positive cells. 15 – 20 neurons were analyzed. Data are shown as mean ± SEM. Unpaired and two-tailed t test (**a**) or one-way ANOVA with Dunnett's multiple comparisons test (**b**), * $p < 0.05$, ** $p < 0.01$, *** $p < 0.001$.

Supplementary Figure 5

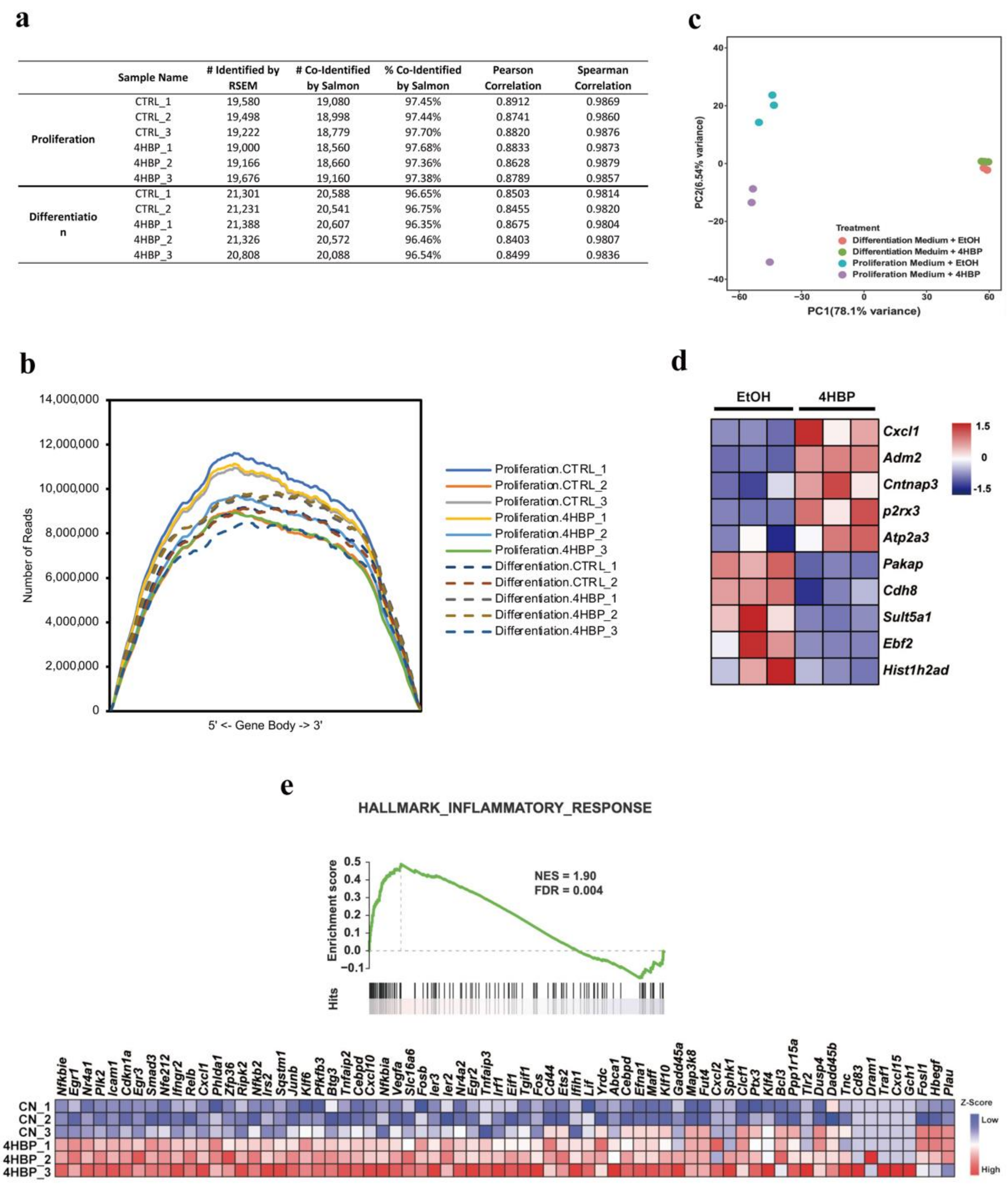

	Sample Name	# Identified by RSEM	# Co-Identified by Salmon	% Co-Identified by Salmon	Pearson Correlation	Spearman Correlation
Proliferation	CTRL_1	19,580	19,080	97.45%	0.8912	0.9869
	CTRL_2	19,498	18,998	97.44%	0.8741	0.9860
	CTRL_3	19,222	18,779	97.70%	0.8820	0.9876
	4HBP_1	19,000	18,560	97.68%	0.8833	0.9873
	4HBP_2	19,166	18,660	97.36%	0.8628	0.9879
	4HBP_3	19,676	19,160	97.38%	0.8789	0.9857
Differentiation	CTRL_1	21,301	20,588	96.65%	0.8503	0.9814
	CTRL_2	21,231	20,541	96.75%	0.8455	0.9820
	4HBP_1	21,388	20,607	96.35%	0.8675	0.9804
	4HBP_2	21,326	20,572	96.46%	0.8403	0.9807
	4HBP_3	20,808	20,088	96.54%	0.8499	0.9836

Supplementary Figure 5. Quality assessment of the transcriptomic analyses. a, The accuracy evaluation of gene expression quantification by RSEM. The Pearson and Spearman correlation coefficients were calculated from the genes co-identified by RSEM and Salmon using the stats R package (v3.6.1). **b**, The gene body coverage statistics at the resolution of 150 bins per gene. **c**, Primary component analysis (PCA)

of all 11 samples using the stats R package (v3.6.1). **d**, Validation of top deregulated genes upon 4HBP treatment under proliferation condition using qPCR. **e**, GSEA plot of HALLMARK_INFLAMMATORY_RESPONSE by draw. GSEA function from NetBID R package (v-2.0.2) and the heatmap of the leading-edge genes of this gene set by pheatmap R package (v1.0.12).

Supplementary Figure 6

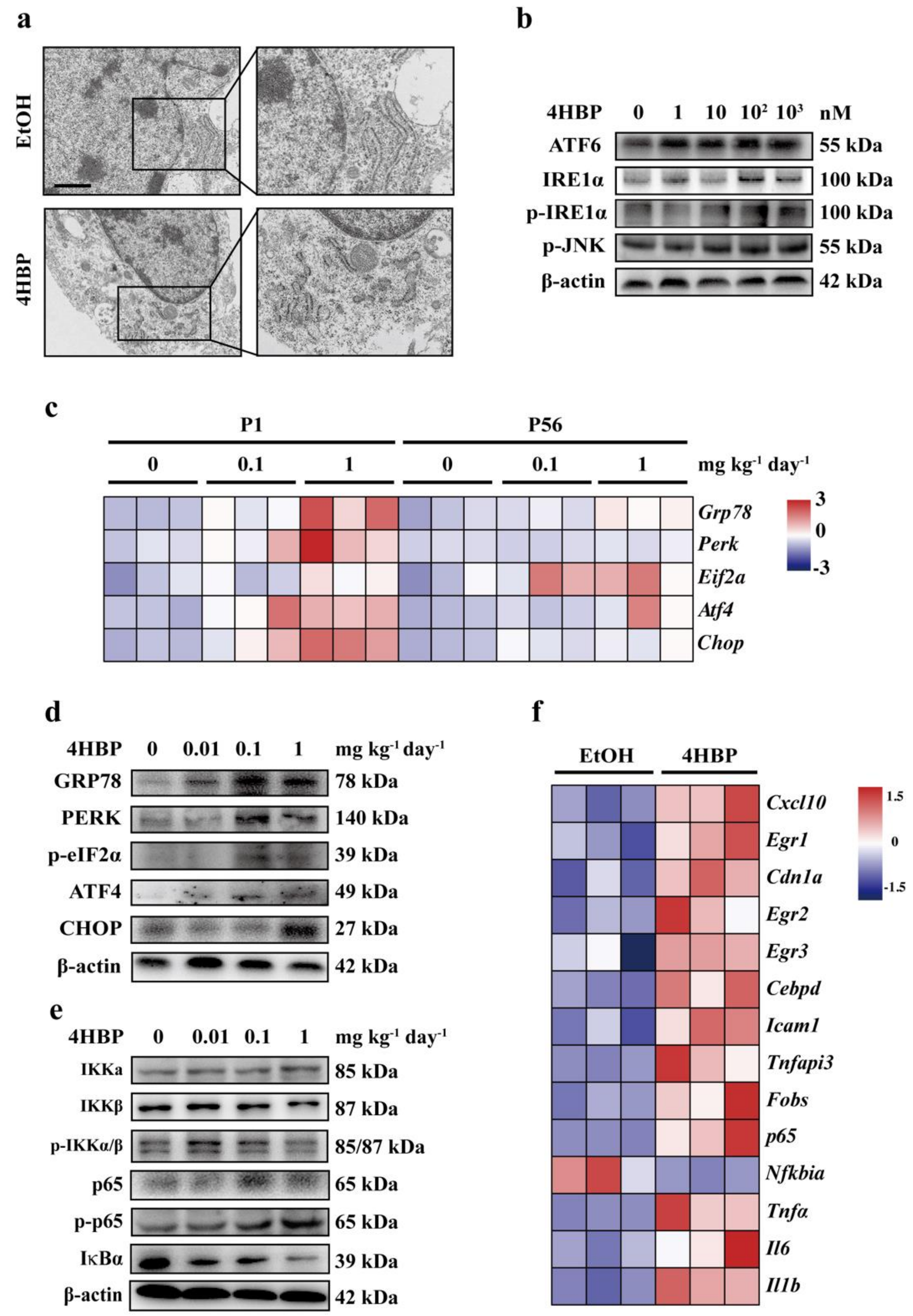

Supplementary Figure 6. Maternal 4HBP exposure induces ER stress and inflammation in offspring hippocampus. **a**, Primary NSCs were exposed to either EtOH (control) or 1 μM 4HBP for 7 days in proliferation media before analyzed by TEM. Scale bar, 2 μm. **b**, Western analyses of ATF6 and IRE1α pathway in

hippocampus-derived NSCs treated with different concentrations of 4HBP for 7 days in proliferation media. **c**, RNA was extracted from hippocampal tissues of P1 and P56 offspring exposed to indicated doses of 4HBP. The levels of key genes in the PERK pathway were detected by qPCR. Three replicates per group. **d**, Western analyses of PERK pathway in the P56 hippocampus. **e**, Western analyses of NFκB pathway in the P56 hippocampus. **f**, RNA was extracted from NSCs treated with either EtOH or 1 μM 4HBP for 7 days in proliferation media. The level of genes in the NFκB pathway and inflammatory cytokines were demonstrated by heatmap. Three replicates per group. Results of Western blot were from a representative experiment in triplicates.

Supplementary Figure 7

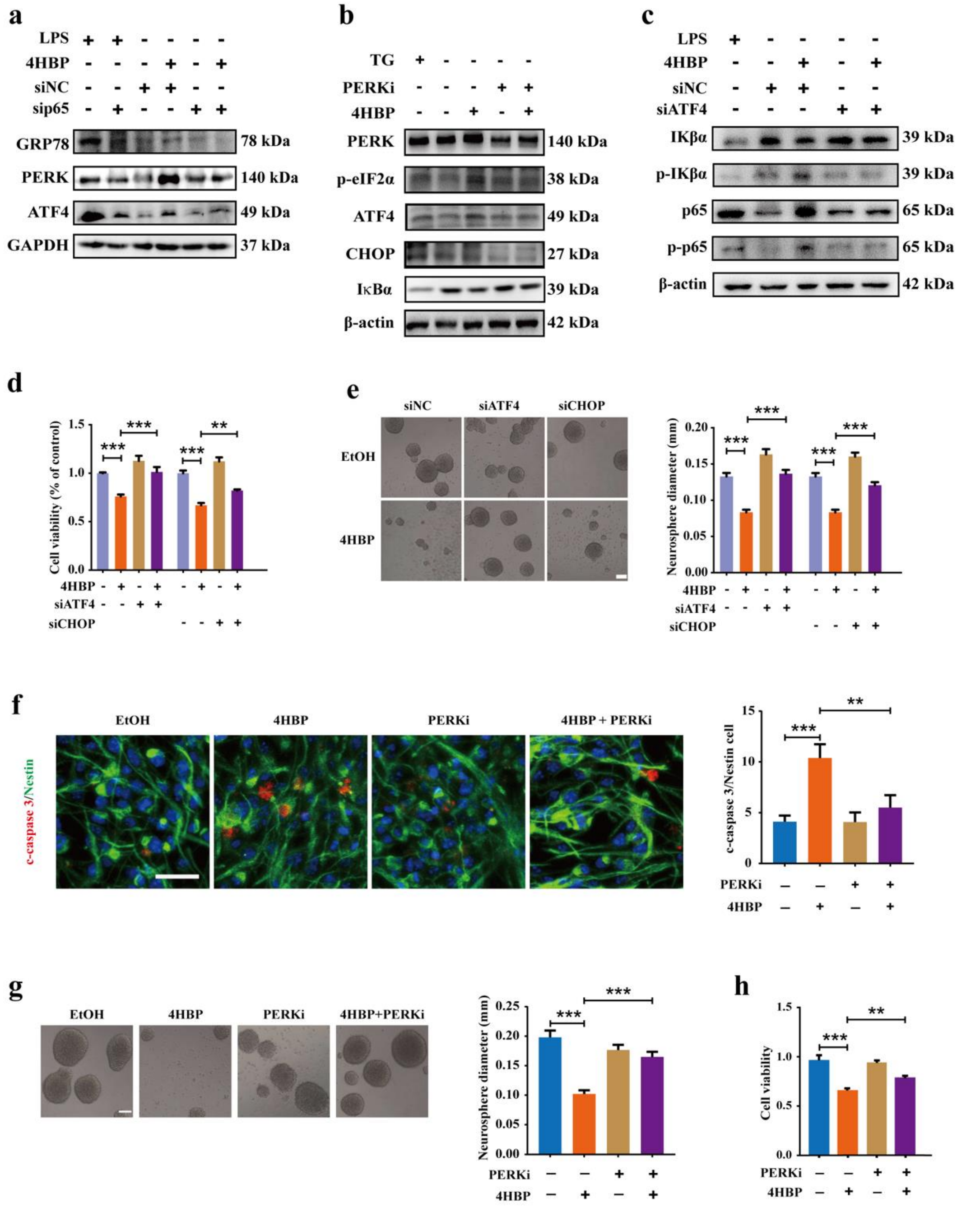

Supplementary Figure 7. PERK-eIF2α axis forms a positive feedback loop with NFκB signaling to promote apoptosis in NSCs exposed to 4HBP. a, Primary NSCs were transfected with either scramble or p65 siRNA followed by 1 μM 4HBP treatment for 7 days in proliferation media. The cells were then harvested for Western

analyses. **b**, NSCs were cultured in the presence of 1 μM 4HBP for 7 days and treated with 0.5 μM GSK2606414 (PERKi) for 12 hours in proliferation media before harvesting cells for Western analyses. TG (200 nM, 2 hours) was used as a positive control. **c**, Primary NSCs were transfected with either scramble or ATF4 siRNA followed by 1 μM 4HBP treatment for 7 days in proliferation media. The cells were harvested for Western analyses. LPS (0.2 μg/ml, 2 hours) was used as a positive control. **d, e**, Primary NSCs were transfected with scramble, ATF4 siRNA and CHOP siRNA followed by 1 μM 4HBP treatment for 7 days in proliferation media. **d**, CCK-8 assay was performed for viability test. **e**, Representative images of neurospheres and quantification of neurosphere diameters (n = 150 – 300 neurospheres in each group). Scale bar, 100 μm. **f**, As in b, representative images and quantification of NSCs co-labeled with Nestin and cleaved-caspase 3. Scale bar, 25 μm. **g**, As in **b,** but NSCs were cultured in suspension to allow for neurosphere formation. Representative images of neurospheres are shown and neurosphere diameter was quantified. Scale bar, 100 μm. **h,** As in **b**, CCK-8 assay was performed to test cell viability. Results of Western blot were from a representative experiment in triplicates. Data are shown as mean ± SEM. One-way ANOVA with Fisher's LSD test, ** $p < 0.01$, *** $p < 0.001$.

Supplementary Figure 8

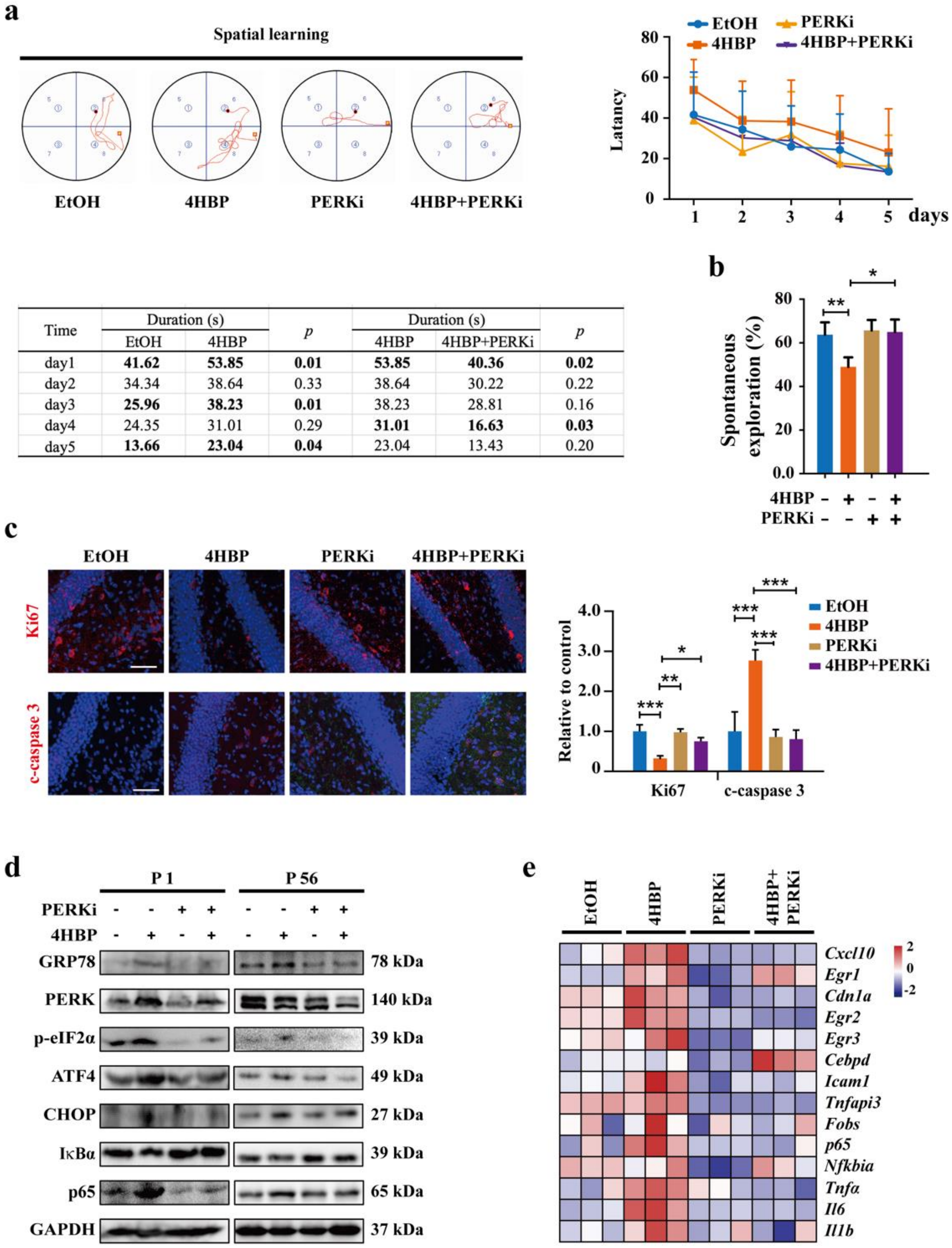

Time	Duration (s)		p	Duration (s)		p
	EtOH	4HBP		4HBP	4HBP+PERKi	
day1	**41.62**	**53.85**	**0.01**	**53.85**	**40.36**	**0.02**
day2	34.34	38.64	0.33	38.64	30.22	0.22
day3	**25.96**	**38.23**	**0.01**	38.23	28.81	0.16
day4	24.35	31.01	0.29	**31.01**	**16.63**	**0.03**
day5	**13.66**	**23.04**	**0.04**	23.04	13.43	0.20

Supplementary Figure 8. PERK blockade improves learning and memory function in offspring mice after maternal 4HBP exposure. Pregnant mice were exposed to 1 mg kg^{-1} day^{-1} 4HBP and treated with or without 50 mg kg^{-1} day^{-1} GSK2606414 (PERKi) via oral gavage throughout the entire pregnancy. The offspring were housed under normal condition until postnatal day 56 for behavioral

tests. **a,** Left, representative swimming trace in the spatial learning test on day 5. Right, escape latency to platform in the spatial learning test. Table shows the statistical analysis results. **b**, Quantification of spontaneous exploration behaviors of mice in the T-maze. Data from n = 8 mice per group. **c**, Left, representative immunofluorescent images of Ki67 and cleaved-caspase 3 in the hippocampus of P56 offspring. Scale bar, 100 μm. Right, Quantification of immunofluorescent images. Data from n = 3 mice per group. **d**, Western blot analyses of proteins in the PERK-ATF4 pathway as well as p65 and IκBα in the hippocampus of P1 and P56 offspring. Results of Western blot were from a representative experiment in triplicates. **e**, The level of genes in the NFκB pathway and inflammatory cytokines are shown by heatmap. n = 3 mice per group. Data are shown as mean ± SEM. One-way ANOVA with Fisher's LSD test, * $p < 0.05$, ** $p < 0.01$, *** $p < 0.001$.

Supplementary Figure 9

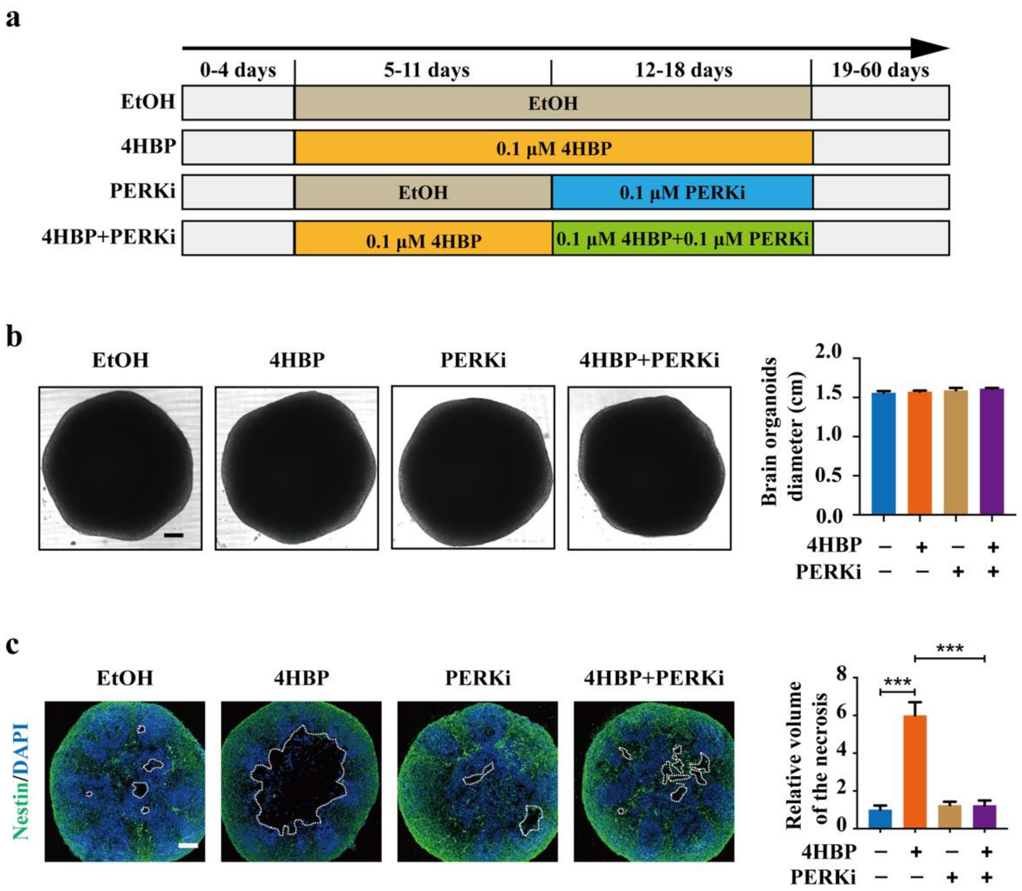

Supplementary Figure 9. PERK inhibition ameliorates the 4HBP-induced neurotoxicity in human brain organoids. a, Schematic overview of human brain organoids culture and treatment. The human brain organoids were exposed to two weeks of 0.5 μM 4HBP, and supplemented with 0.1 μM GSK2606414 (PERKi) for 1 week, then cultured and allowed for organoid growth for 2 months. **b**, Representative morphological images of human brain organoids and quantification of organoid size on day 60. Scale bar, 200 μm. Three organoids per group. **c**, Representative images and quantification of necrosis area (marked in dashed line) in the human brain organoids. Scale bar, 100 μm. Three organoids in each group and 3 or 4 visual fields

for each organoid. Data are shown as mean ± SEM. One-way ANOVA with Fisher's LSD test, *** $p < 0.001$.

Supplementary Tables

Primer Name	Forward (5'-3')	Reverse (5'-3')	Application

Table S1. Primers used in this study.

Grp78	CCGAGGAGGAGGACAAGAAGGAG	GAACACACCGACGCAGGAATAGG	qPCR
Perk	GGACGAATCGCTGCACTGGATG	GGCTTGCTGAGGCTAGATGAAACC	qPCR
Eif2a	GCATTCTTCGCCATGTTGCTGAG	TAGGCACCGTATCCAGGTCTCTTG	qPCR
Atf4	TCTGCCTTCTCCAGGTGGTTCC	GCTGCTGTCTTGTTTTGCTCCATC	qPCR
chop	CCCTCGCTCTCCAGATTCCAGTC	TCGTTCTCCTGCTCCTTCTCCTTC	qPCR
Ire1a	ACACCGACCACCGTATCTCAGG	GAACGCCACCCATCCAACCAG	qPCR
Xbp1	TTGGGCATTCTGGACAAGTTGGAC	ACAGAGAAAGGGAGGCTGGTAAGG	qPCR
JNK	CGCCTTATGTGGTGACTCGCTAC	CTCCCATGATGCACCCAACTGAC	qPCR
Xbp1s	TGAGAACCAGGAGTTAAGAACACGC	CCTGCACCTGCTGCGGAC	qPCR
Atf6	TCGGTCAGTGGACTCTTATT	CCAGTGACAGGCTTATCTTC	qPCR
Tnfa	AAATGGGCTCCCTCTCATCAGTTC	TCTGCTTGGTGGTTTGCTACGAC	qPCR
Il6	TCCTACCCCAACTTCCAATGCTC	TTGGATGGTCTTGGTCCTTAGCC	qPCR
Il1b	CACCTCTCAAGCAGAGCACAG	GGGTTCCATGGTGAAGTCAAC	qPCR
Cxcl1	CCGCTCGCTTCTCTGTGCAG	ACTGACAGCGCAGCTCATTGG	qPCR
Adm2	TCGGTTGCATCAGCCTCCTCTAC	ACGACGAGACTTCCAGACTACAGG	qPCR
Cntnap3	TGTGATGCTCCGTTGGCTTCTTC	GCAGTGACCTCCATTCGTTCTCC	qPCR
P2rx3	GGATCATCAACCGAGCCGTTCAG	CTCTGTTGGCATAGCGTCCGAAG	qPCR
Atp2a3	CATTGTGCGGAGCCTGCCTTC	GCTTCAGCCACCACGAACATCC	qPCR
Pakap	CACTGCCTTCCTTGCCGATGAC	GACCTGGTTGACCACGATGACTTC	qPCR
Cdh8	TGCTGCTCGTCATTGTGGTTCTG	TCCTCCTTCGTCGTCGTAGCG	qPCR
Sult5a1	CTGGATGCAGCAGGTTCTGAGTC	CCGCTTGGAATGGAGGCTATCG	qPCR
Ebf2	GCGGTTCCAGGTCGTGTTGTC	GCCTTCTTGCTCTCCTTCCATGC	qPCR
Hist1h2ad	GACAACAAGAAGACGCGCATCATC	CTTGGCCTTGTGGTGGCTCTC	qPCR
GAPDH	GTCAGTGGTGGACCTGACCT	TCGCTGTTGAAGTCAGAGGA	qPCR
P65-P1	GCCCCCTGCCAAAAGTAAAC	GACTTGTTTCCCAGGCTCTGA	ChIP
P65-P2	TCAGAGCCTGGGAAACAAGTC	GGGCGGAGTCTTCTCCAGAT	ChIP
P65-P3	GCCTGCTGATTCAAGTGTCC	TAAATCCCGGAGCCTCGTCT	ChIP
P65-P4	GTGACATCACCAAACTCCGC	GGGTCCGCCGATTACTCAC	ChIP
